# A plasmonic meta-rotary travelling-wave oscillator with ultrahigh phase accuracy and figure of merit

**DOI:** 10.1038/s41377-025-01966-z

**Published:** 2025-08-21

**Authors:** Da Yue Yao, Hao Chi Zhang, Pei Hang He, Jia Jie Shen, Jia Wen Zhu, Peigen Zhou, Xin Yu Zhang, Le Peng Zhang, Li Jie Wu, Cun Yue Wei, Rui Wen Shao, Yi Fan, Yang Zhao, Jixin Chen, Wei Hong, Tie Jun Cui

**Affiliations:** 1 State Key Laboratory of Millimeter Waves, Southeast University, Nanjing, China; 2 Institute of Electromagnetic Space, Southeast University, Nanjing, China

**Keywords:** Slow light, Sub-wavelength optics, Metamaterials

## Abstract

High phase accuracy and figure of merit (FOM) of quadrature signals are essential for integrated systems, including quadrature amplitude modulation (QAM) communications and multi-input multi-output (MIMO) radar. However, the traditional quadrature oscillators often struggle to meet the stringent requirements of high FOM and high quadrature phase accuracy simultaneously. To address this challenge, we propose a spoof surface plasmon polariton (SPP) metawaveguide (Meta) to design on-chip rotary traveling-wave oscillator (RTWO). By leveraging the advanced dispersion manipulation capability of Meta, the physical and electrical lengths of transmission line (TL) are effectively decoupled, thereby overcoming the limitations associated with the unequal electrical lengths of inner and outer loops of the resonator, which is difficult to achieve in the conventional RTWOs. Based on the design methodology, we realize a Meta-RTWO using the 65 nm CMOS technology and achieve a measured FOM of 188.5 dBc/Hz and a phase error of approximately 0.21°. These metrics surpass those of the traditional oscillators fabricated even by more advanced 28 nm CMOS processes. This study demonstrates that Meta-RTWO achieves a significant improvement in both output signal quadrature accuracy and FOM under process limitations without using additional phase adjustment structures.

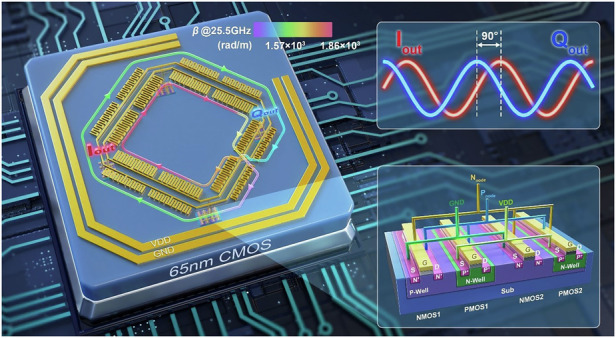

## Abstract (web summary)

**Meta-RTWO based on spoof SPPs**. A meta-RTWO chip is proposed with ultrahigh phase accuracy and figure of merit based on spoof surface plasmon polaritons.

## Introduction

On-chip quadrature resonant oscillators represent a special category of the resonant oscillators that can simultaneously generate two quadrature signals without using additional chip area^1^. These oscillators are extensively used in integrated systems, including quadrature amplitude modulation (QAM) communications^2^ and multi-input multi-output (MIMO) radar^3^. From a performance standpoint, the figure of merit (FOM) of the oscillator and the phase accuracy of output quadrature signals are the most critical parameters for evaluating the overall efficacy. FOM serves as a comprehensive indicator of the power consumption, frequency stability, and phase noise (PN) characteristics of the oscillator, and subpar performance typically indicates the PN deterioration. Deficiencies in phase accuracy can result in the generation of spurious mirror frequencies, ultimately leading to increase of bit error rate (BER)^4^. In the conventional architectures, LC oscillators represent a predominant category of on-chip quadrature resonant oscillators owing to their inherently high quality (Q) factor, which facilitates the attainment of superior PN performance. Specifically, the LC oscillators are used to generate the quadrature signals through various techniques, including RC polyphase filtering (PPF), frequency divider and coupled quadrature oscillator. Among these methods, the PPF approach excels to achieve a high quadrature phase accuracy through multilevel network superposition. However, this technique incurs trade-offs, which are manifested as the increases in both chip area and system insertion loss^5^. The frequency divider method can effectively mitigate the push/pull effects exerted by robust power amplifiers in transceiver systems on the oscillator source^6^. Nonetheless, this approach necessitates a high-speed frequency divider, which consequently leads to an elevated power consumption and a significant increase in the occupied area.

The coupled quadrature oscillator method is widely adopted to generate the quadrature signals in the microwave band^7,8^. In this method, two interconnected identical oscillators are employed to produce the quadrature signals, offering advantages such as a low power consumption and reduced PN. The coupling methods^9^ include series coupling^10^, in-phase coupling^11,12^, superharmonic coupling^13,14^, transformer coupling^15,16^, and phase-locked loop coupling^17^. However, the coupling between two identical oscillators forces the oscillation frequency to be shifted from the resonant frequency of the LC resonance, which reduces the Q factor of the resonator and thus deteriorates the PN of the oscillator. In particular, the increase in the coupling strength of the coupling structure enhances the control of the signal quadrature and improves the quadrature phase accuracy. However, this increase also results in a larger offset of frequency, which can degrade the PN performance. Conversely, a decrease in the coupling strength leads to frequency mismatch between two identical oscillators, resulting in significant quadrature phase errors. Thus, this configuration faces a trade-off between the PN and quadrature phase accuracy^18^. Additionally, all coupling methods require supplementary phase adjustment mechanisms to achieve high-precision quadrature signals^13,17^. However, the introduction of these phase adjustment structures can increase the power consumption and further degrade PN.

As a distributed oscillator, the rotating traveling-wave oscillator (RTWO)^19–23^ has the advantages of low jitter and low PN of microwave frequency signals and can naturally realize multiphase signal outputs without using additional structures^24^ and alleviate the problem of index trade-off between the PN of the coupled quadrature oscillator and the quadrature phase accuracy. In general, an RTWO consists of a head-to-tail Möbius ring resonator and uniformly distributed negative resistance (-*Gm*) cells (see Fig. 2a). However, the planar topological characteristics of chip process lead to an inherent imbalance between the inner and outer rings of the Möbius ring resonator, which makes it difficult to align the phases of the currents injected into the resonance cavity by individual -*Gm* cells of RTWO, leading to high phase errors and high PN. The conventional approaches typically employ varactor diodes within resonator structures to realize RTWO phase adjustment^1^. However, the inherent low Q factor of CMOS integrated varactors at high frequencies induced significant PN degradation in the RTWO implementations.

To address this issue, a new degree of freedom derived from fundamental physical principles must be introduced, specifically by regulating the phase constants (*β*) of waves to decouple the electrical length from the physical length, thereby enabling RTWO to achieve both high phase accuracy and high FOM. For this goal, we propose a slow-wave (SW)^25–27^ metawaveguide (Meta) to design RTWO via spoof surface plasmon polaritons (spoof SPPs)^28–40^. The spoof SPPs are classified as a type of metamaterials, in which intentionally designed and arranged periodic metallic structures are used to replicate the physical properties of optical SPPs^41–43^. A detailed comparison of spoof SPPs with other slow-wave structures and metamaterial transmission lines can be found in “Introduction to other metamaterial transmission lines and slow-wave structures” of the Supplementary information. The spoof SPPs enable continuous regulations of electromagnetic waves through adjusting the metal structure dimensions, offering advantages such as tuneable dispersion characteristics, enhanced field confinement, and slow-wave properties. In short, in addition to reproducing the properties exhibited by SPPs in the microwave band, the spoof SPP structure provides a practical solution for controlling the dispersion properties via structural design and a direct means for designing the transmission behavior of electromagnetic waves.

**Fig. 1:**
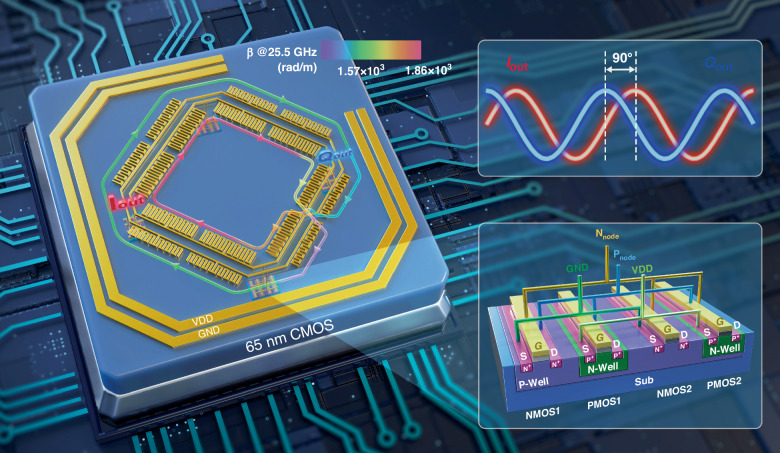
Conceptual diagram of Meta-RTWO based on spoof SPP

Here, we present the design of Meta-RTWO by introducing a spoof SPP structure into the resonator at the chip scale, as shown in Fig. 1. This approach addresses the challenge faced by the traditional oscillators in meeting the requirements of high FOM and high quadrature phase accuracy simultaneously when generating the quadrature signals. By using the flexible capability to control the dispersion property of spoof SPPs, we can optimize both phase accuracy and PN of the output signal without introducing any additional coupling structures for adjustment. The Meta-RTWO chip is fabricated using the standard 65 nm CMOS process, and the measured oscillation frequency is 25.5 GHz with the phase error of approximately 0.21°. The measured PN is -112.7 dBc/Hz at 1 MHz, whereas the core power consumption is 17.4 mW, resulting in an FOM of 188.5 dBc/Hz.

## Results

### Theoretical analysis of Meta-RTWO

**Fig. 2:**
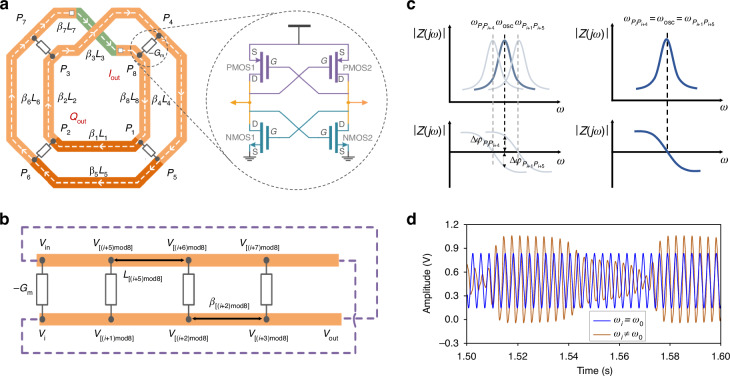
**Theoretical analysis of Meta-RTWO**. **a** The Meta-RTWO architecture and the classical *-Gm* cell structure. **b** The circuit of Meta-RTWO after decoupling. **c** Curves of amplitude versus phase response of the resonator. **d** The time-domain simulation outputs of Meta-RTWO in the cases of *ω*_*i*_ = *ω*_0_ = 4.7e^11^rad/s and *ω*_*i+*4_ − *ω*_*i*_ = 1.7e^11^rad/s

The classical design of RTWO features a head-to-tail Möbius ring resonator, along with uniformly distributed *-Gm* cells, as illustrated in Fig. 2a. The traveling-wave signal undergoes cyclic propagation along the Möbius ring, establishing a self-closed feedback path. The *-Gm* cells are usually composed of two cross-coupled inverters that provide energy compensation in the circuit while ensure odd modes between the inner and outer rings. Each inverter consists of an N-channel metal-oxide-semiconductor field effect transistor (N-MOSFET, NMOS) and a PMOS, with the transconductances denoted as *gm*_*n*_ and *gm*_*p*_, respectively, connected in series. Please refer to “Parasitic effects of *-Gm* cells on wave propagation behavior” in Supplementary information for details. In RTWO operation, a steady resonant state, characterized by the phase of the wave along TL being a periodic function of its position, must be maintained while the amplitude remains constant. This configuration allows to generate multiple signals with a constant phase difference from various locations. In analyzing the oscillatory behavior of RTWO, one can start from the following three conditions: the starting mechanism from the Barkhausen oscillation condition, the conditions for stable oscillation and low PN from the multi-core standing-wave oscillator (SWO) coupling, and the conditions for obtaining a high phase accuracy from the wave transmission characteristics. For an RTWO to achieve both high phase accuracy and low PN, these three conditions must be satisfied simultaneously.

#### Meta-RTWO startup conditions

The oscillator startup conditions are typically analyzed by the Barkhausen criterion, which treats the oscillator as a closed negative feedback system. The relationship between the input and output signals of the system is described by transfer function *H*(*ω*). To start the oscillation, the output signal of the system, after passing through the feedback loop to the input signal, can continuously enhance and maintain the oscillation, which requires the transfer function to satisfy *H*(*ω*) > 1 (amplitude balance condition) and ∠*H*(*ω*)=2π (phase balance condition). The former provides continuous gain to the signal and the latter ensures that the feedback signal and original signal are superimposed in the same phase. To examine the transfer function of Meta-RTWO, we conduct a decoupling operation on the RTWO model, as shown in Fig. 2b. RTWO depicted in Fig. 2a can be conceptualized as a distributed oscillator composed of four *-Gm* cells. When observed from the terminals of any -*Gm* cell, RTWO can be considered as a network of the input *V*_in_ and output *V*_out_ of a fully differential distributed amplifier^44,45^ connected together. The derivation of the Meta-RTWO system function is described in the Supplementary information “Derivation of Meta-RTWO startup conditions”. The amplitude and phase balance conditions to be satisfied for RTWO vibration are derived as

1 $$\left\{\begin{array}{c}{(1-{G}_{m}{Z}_{0})}^{4}{{\rm{e}}}^{-\alpha (\omega )L}\ge 1\\ {{\rm{e}}}^{-{\rm{j}}B}=-1\Rightarrow B=\pi \end{array}\right.$$

where

$$B=\beta {(\omega )}_{i}{L}_{i}+\mathop{\sum }\limits_{j=1}^{3}\beta {(\omega )}_{[(i+j)\mathrm{mod}\,8]}{L}_{[(i+j)\mathrm{mod}\,8]}$$

In general, the amplitude balance condition can be satisfied by designing the size of the MOS -*Gm* cells and changing the Gm value. From Eq. (1), the phase balance condition required for oscillation can be further deduced as:

2 $$\left\{\begin{array}{l}\mathop{\sum }\limits_{i=1}^{8}\beta {(\omega )}_{i}{L}_{i}=2\pi \\ \beta {(\omega )}_{i}{L}_{i}=\beta {(\omega )}_{i+4}{L}_{i+4},i=1,2,3,4\end{array}\right.$$

Hence the RTWO oscillation requires that the total electrical length of the resonator is equal to 2π, and the electrical lengths of inner and outer loop TLs connecting the negative resistance unit are equal.

#### Stability analysis of Meta-RTWO

Using a similar methodology (see “Analysis of RTWOs compared to multi-core SWOs” in Supplementary information for details), the solution to the dynamic equations of the Meta-RTWO system can be expressed as: *β(ω*_*i*_*)*_*i*_*L*_*i*_ = π/4, *i* = 1,2,…,8. When the electrical lengths of each segment of TL at the same frequency are equal to π/4, i.e.,

3 $$\beta {({\omega }_{0})}_{i}{L}_{i}=\frac{\pi }{4},i=1,2,\cdots ,8$$

the four SWOs are operated at the same oscillation frequency. At this point, Meta-RTWO achieves an ideal stable state. When each segment of the TL satisfies a small difference in *ω*_*i*_ with an electrical length equal to π/4, the four SWOs are forced to be injection locked to a common frequency for oscillation, as shown in Fig. 2c. This means that some SWOs deviate from their resonant frequencies and the effective Q factor of the energy storage circuit decreases, resulting in worse PN. When each segment of TL satisfies a large difference in *ω*_*i*_ with an electrical length equal to π/4, the resonant frequencies of individual SWOs are more divergent, the injection locking is relaxed, and the system starts to oscillate at different frequencies. The simulation results validate the preceding conclusion. The time-domain simulation outputs in both cases *ω*_*i*_ = *ω*_0_ = 4.7e^11^rad/s and *ω*_*i+*4_ − *ω*_*i*_ = 1.7e^11^rad/s are presented in Fig. 2d. The signal envelopes reveal that RTWO remains unlocked and is oscillated in an unstable state when the *ω*_*i*_ differences are substantial.

#### Phase error analysis of Meta-RTWO

The analysis of the phase error in Meta-RTWO is conducted from the perspective of the wave propagation characteristics. The waves propagate as traveling waves in the Meta-RTWO resonator, allowing natural realization of multiphase signal outputs. To obtain an accurate quadrature signal output, the following conditions must be satisfied:

4 $$\beta {(\omega )}_{[(i+2){\rm{mod}}\,8]}{L}_{[(i+2){\rm{mod}}\,8]}-{\beta }_{i}{L}_{i}=\frac{\pi }{2},i=1,2,\cdots 8$$

For RTWO, the three aforementioned conditions must be concurrently satisfied to achieve the quadrature signal outputs with a high phase accuracy and minimal PN. By solving Eqs. (2)–(4), the resulting solution is obtained, as presented in Eq. (3). Specifically, when the lengths of each segment of TL are equal at the *ω*_0_ frequency, RTWO can produce the signals characterized by high quadrature phase accuracy and FOM.

In the conventional RTWO resonators consist of microstrip transmission-lines (MS TLs), the phase constants of all segments equal at the same frequency, denoted as *β*_*i*_(*ω*_*i*_)=*β*_0_(*ω*_0_), *i* = 1,2,…8. For the ideal steady-state condition in Eq. (3) to hold, the physical lengths of all segments must also be equal, i.e., the condition *L*_*i*_ = *L*_0_(*i* = 1,2,…8) must be fulfilled. However, owing to fabrication constraints, the ultra-thick metal layer used for resonator alignment is limited to the top layer. These processing limitations require the adoption of straight or 135° diagonal paths for TL, resulting in the actual machining layout of the RTWO resonator, as depicted in Fig. 2a. The RTWO resonator comprises a large square and a small square, forming a Möbius ring, with the cross-sections connected by vias to the sub-top layer to complete the jumper. According to the figure, the lengths of the inner and outer rings of RTWO are unequal (*L*_*i*_ ≠ *L*_0_ (*i* = 1,2,…8)), and the cross-jumper segments will introduce an additional imbalance. Thus, the planar topology inherent to the chip fabrication process complicates the alignment of physical lengths of the segments in the RTWO Möbius ring resonator.

Therefore, when the phase constants of each segment in the conventional RTWO resonator are equal but the physical lengths are unequal, there is a pressing need for new technological approaches to modulate the dispersion characteristics of electromagnetic waves. Such advancements would facilitate the decoupling of electrical and physical lengths, which ultimately enables the realization of RTWOs with high FOM and improved quadrature phase accuracy.

### Design of Meta-RTWO

**Fig. 3:**
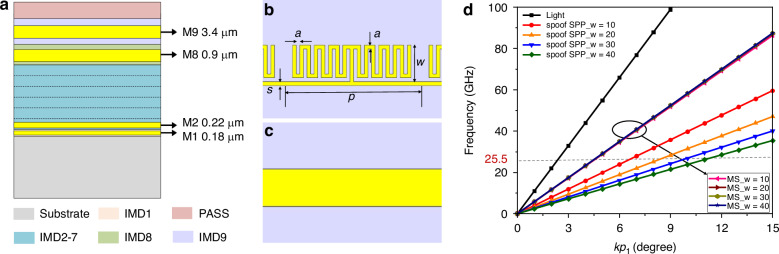
**The design of spoof SPP unit**. **a** The 65 nm CMOS process cross-section. **b** The structure of spoof SPP unit. **c** The corresponding MS structure. **d** The dispersion curves of the light, spoof SPP TLs and MS TLs

We propose to incorporate the spoof SPP Meta into the design of RTWOs. Research indicates that spoof SPP Meta can directly modulate the propagation constants by tuning the geometric parameters, which may address the challenge to achieve a stable quadrature signal output with high FOM and phase accuracy in RTWO constrained by the area limitations. In this design, we use the standard 65 nm CMOS process to implement the spoof SPP TL, with the process cross-sectional parameters illustrated in Fig. 3a (see “The process details of 65nmCMOS” in Supplementary information for details). To minimize the TL losses, the top metal layer is selected as the structural layer for the spoof SPPs, while the jumper segments are connected by vias to the sub-top metal layer. The M1 and M2 layers serve as the grounding layers. Additionally, an MS-RTWO with identical configuration is established as a reference, in which both structures share the same dimensions, and are only different in the TL employed in the resonator.

#### Design of the Meta-RTWO resonator

The conventional spoof SPP structure integrates a central stripline with periodically arranged open-circuited stubs (see “The dispersion analysis of the spoof SPPs” in Supplementary information for more details). To achieve sufficient tuning capability of dispersion at millimeter-wave frequencies, long stubs are usually demanded in the traditional implementations, which is a serious waste of chip area. To reduce the chip size, we develop a miniaturized spoof SPP topology, as shown in Fig. 3b, which comprises a primary strip with a periodically meander open-circuit stub adjacent to its side. The corresponding MS structure is depicted in Fig. 3c. In this design, to realize both high modulation capability and compact on-chip area, the unit size of the spoof SPP TL is given by: *p* = 76 µm, *s* = 2 µm, *a* = 2 µm, and *N* = 4, and the radial length (*w*) of the stub line is adjusted to finely modulate *β* of the electromagnetic wave. The dispersion curve visually illustrates the ability of the spoof SPPs to regulate *β* of electromagnetic waves (see “The dispersion analysis of the spoof SPPs” in Supplementary information for details). As shown in Fig. 3d, the spoof SPP units with various *w* values exhibit distinct dispersion curves, indicating the differences in *β* for the spoof SPP units of equal length at a fixed frequency. In contrast, the dispersion curves of the MS TLs across different *w* values largely overlap. These results highlight the strong capability of spoof SPPs to control the dispersion, which enables the decoupling of electrical and physical lengths along TL. Furthermore, the dispersion curves of the spoof SPP units consistently lie to the right of those of TLs, indicating the slow-wave characteristics. Consequently, the dimensions of the spoof SPPs required to achieve the same electrical lengths are smaller than those of the MS TLs, suggesting that the Meta-RTWO possesses significant potential for miniaturization.

**Fig. 4:**
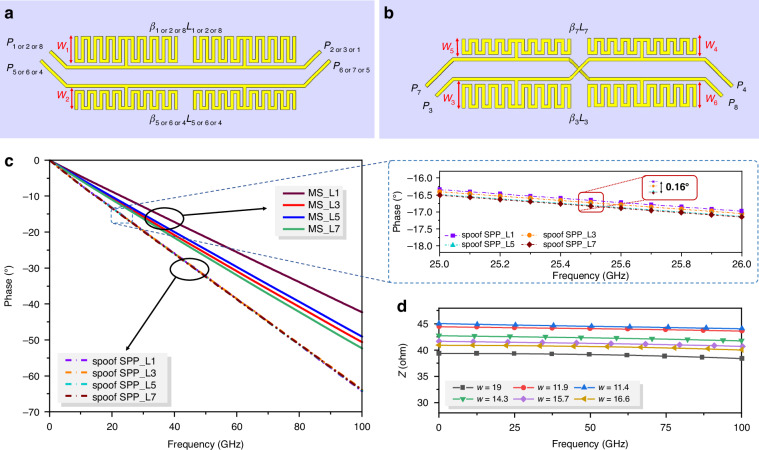
**Design of the Meta-RTWO resonator**. **a** The structure of uncrossed spoof SPP TL (*L*_1_, *L*_2_, *L*_8_, *L*_5_, *L*_6_, and *L*_4_). **b** The structure of crossed jumpered spoof SPP TL (*L*_3_ and *L*_7_). **c** The phase-frequency curves of Meta-RTWO and MS-RTWO resonators with different lengths. **d** Characteristic impedance extraction of different spoof SPP units that make up the Meta-RTWO resonator

Based on the topology shown in Fig. 2a, the Meta-RTWO resonator can be constructed from a three-sided TL formed by replicating the rotation of uncrossed spoof SPP structure (see Fig. 4a) and a one-sided cross-jumper spoof SPP TL (see Fig. 4b) arranged head-to-tail. Since the physical length of the inner circle of the uncrossed spoof SPP TL is shorter than that of the outer circle (*L*_1or2or8_ < *L*_5or6or4_), *w*_1_ of the inner circle of the spoof SPP TL is appropriately increased (see “Design approach for Meta-RTWO resonator” in Supplementary information for details). In addition, because the two wires of the cross-jumper spoof SPP TL span both inner and outer loops, the four corresponding spoof SPP units must be individually designed to satisfy *β*_3_*L*_3_ = *β*_7_*L*_7_ = *β*_*i*_*L*_*i*_. The scattering parameters of TLs are simulated via the time-domain solver in commercial software CST Microwave Studio, with the dimensions of the four TLs optimized to ensure that Meta-RTWO strictly adheres to Eq. (3) at the same frequency. The phase curves of the four spoof SPP TLs for the case of *w*_1_ = 19μm, *w*_2_ = 14.3μm, *w*_3_ = 16.6μm, *w*_4_ = 11.9μm, *w*_5_ = 11.4μm, *w*_6_ = 15.7μm, along with those of four MS TLs of equal lengths, are presented in Fig. 4c.

We observe that the phase curves of spoof SPPs nearly overlap in the frequency range of 0-100 GHz, with a maximum phase difference of only 0.16° at 25.5 GHz. This result indicates that the spoof SPPs effectively satisfy Eq. (3) across various physical lengths. In contrast, the phase differences among the four MS TLs are notable, with a maximum phase difference of 2.81° at 25.5 GHz. The phase difference of the spoof SPP TLs is only 5.6% of that of the MS TLs. The characteristic impedance of different spoof SPP units are extracted via the method outlined in ref.^46^ (see Methods for details), revealing that the characteristic impedance of the spoof SPP TL remains largely unchanged with variations in *w*, as illustrated in Fig. 4d. At 25.5 GHz, the maximum difference in the characteristic impedance between neighboring spoof SPP TLs is 2.1Ω, resulting in the reflected energy proportion of approximately 0.5%, which is negligible. The analysis of the transmission loss in spoof SPP TL is detailed in “Analysis of the loss in spoof SPP TL and the efficiency of Meta-RTWO” in the Supplementary information.

#### Design of Meta-RTWO chip and experimental validation

**Fig. 5:**
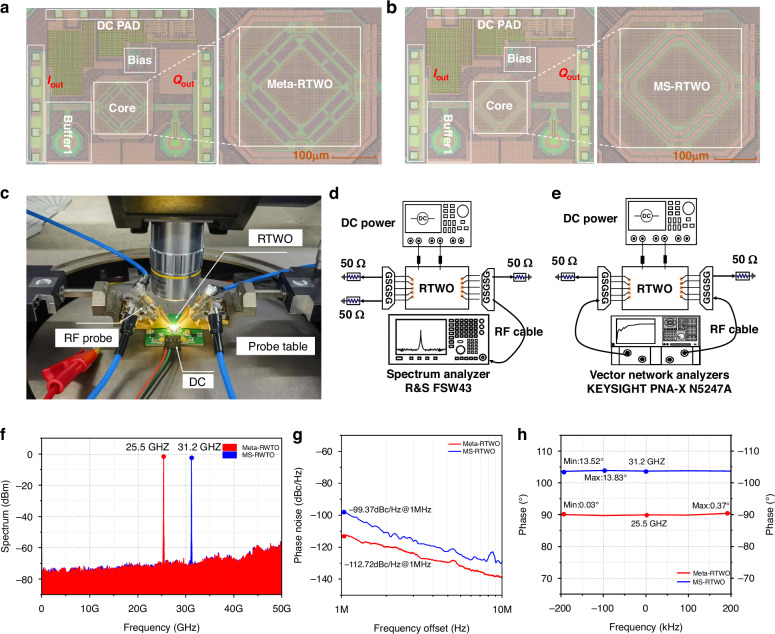
**Experimental validation**. **a** Microscope picture of the Meta-RTWO chip. **b** Microscope picture of the MS-RTWO chip. **c** Chip measurement field. **d** Test block diagram of the spectrum and PN sum measured by the spectrum analyzer. **e** Test block diagram of the phase error measured by VNA. **f** Measured results of the spectrum for Meta-RTWO and MS-RTWO. **g** Measured results of PNs for Meta-RTWO and MS-RTWO. **h** Measured results of quadrature signal phase errors for Meta-RTWO and MS-RTWO

We fabricate the Meta-RTWO chip and its corresponding MS-RTWO (see “RTWO circuit structure” in the Supplementary information for details) counterpart using a 65 nm CMOS process, as illustrated in Fig. 5a, b. To maintain the circuit balance, two differential quadrature signals (S1 and S2) are routed from the left and right -*Gm* cell nodes of the resonator. The overall chip dimension is 1150 × 950 µm, with the core oscillator module occupying approximately 0.051 mm². Simulation results indicate that the RTWO oscillation startup time is in the nanosecond scale (see “Analysis of RTWO Startup time” in the Supplementary Information for details). The comprehensive performance testing of the chip is conducted via on-chip probe measurements, as shown in Fig. 5c.

Typically, FOM of an oscillator is indirectly derived from its power consumption, frequency, and PN as

5 $$F{\rm{OM}}(\Delta f)=-PN(\Delta f)+20{\log }_{10}(f/\Delta f)-10{\log }_{10}({P}_{DC}/1{\rm{mW}})$$

The test system comprises a spectrum analyzer, a DC power supply, and a matched load, with the connection diagram presented in Fig. 5d. Detailed information can be found in the Methods section. For power consumption, with the chip supply voltage fixed at 1 V, the total current of Meta-RTWO is measured as 45 mA, whereas the core current of the oscillator is approximately 17.4 mA, resulting in a core power consumption of approximately 17.4 mW. In comparison, the MS-RTWO exhibits a total current of 59 mA, with a core current of approximately 22.7 mA and a core power consumption of approximately 22.7 mW. With respect to frequency measurements, we observe the output spectra of signals S1 from both Meta-RTWO and MS-RTWO, as shown in Fig. 5f. The Meta-RTWO is operated at 25.5 GHz with an output power of −1.36 dBm, whereas the MS-RTWO is operated at 31.2 GHz with an output power of −2.20 dBm. After accounting for the probes and cables at these frequencies, the actual output power of Meta-RTWO is adjusted to 2.2 dBm, and the corresponding power of MS-RTWO is corrected to 1.91 dBm. These results indicate that Meta-RTWO is operated at a slightly lower frequency than MS-RTWO for the same chip area, which is attributed to the larger phase constant of spoof SPP TLs. Consequently, the dimensions required for spoof SPP TLs to achieve the same electrical length are smaller than those for MS TLs, highlighting the potential for miniaturization in the Meta-RTWO design.

For PN, we further evaluate the output signals S1 of Meta-RTWO and MS-RTWO using a spectrum analyzer, yielding the PN levels of -112.7 dBc/Hz@1 MHz and -99.4 dBc/Hz@1 MHz, respectively, as shown in Fig. 5g. Substitution of these values into Eq. (5) allows us to compute FOMs for Meta-RTWO and MS-RTWO, which are 188.5 dBc/Hz and 175.7 dBc/Hz, respectively. These results suggest that Meta-RTWO based on spoof SPPs achieves much lower PN while maintains the equivalent power consumption and frequency. To determine the phase difference between two output signals of RTWO (see “Signal phase difference measurement method” in Supplementary information for details), experiments are conducted in the receiver mode of a vector network analyzer (VNA), as detailed in the Methods section. The test block diagram is presented in Fig. 5e. The phase difference results for the Meta-RTWO and MS-RTWO are illustrated in Fig. 5h. The mean value of the phase errors for the Meta-RTWO is measured as 0.21°, whereas that for MS-RTWO is approximately 13.68°, indicating that the error of the former is negligible in practical applications.

A comparative analysis of the Meta-RTWO performance against that of other quadrature oscillators reported in recent years is summarized in Table 1. The proposed Meta-RTWO exhibits an exceptionally high FOM with a minimal quadrature phase error. To the best of our knowledge, these results represent the highest performance in terms of measured quadrature phase accuracy and FOM metrics without the need for additional phase adjustment structures. Notably, the works with similar metrics to those in this paper (e.g., refs. ^18,20^.) required a 28 nm CMOS process, which is three generations more advanced and more expensive than the 65 nm CMOS process used in this work, and is inherently more likely to achieve better noise performance and better phase accuracy. Hence the Meta-RTWO achieves a double breakthrough in the output signal quadrature accuracy and PN metrics under the process constraints and without any additional phase adjustment structures.

**Table 1:** Performance summary and comparison with the state-of-the-art

Reference	JSSC’24 [17]	JSSC’19 [13]	JSSC’23 [15]	JSSC’20 [16]	TMTT’13 [47]	JSSC’18 [8]	TMTT’15 [11]	JSSC’18 [14]	JSSC’15 [12]	JSSC’21 [20]	JSSC’18 [21]	TCAS I’18 [22]	JSSC’12 [23]	Comparison object	This work
Technology	28 nm CMOS	28 nm CMOS	28 nm CMOS	45 nm SOI	65 nm CMOS	65 nm CMOS	65 nm CMOS	40 nm CMOS	65 nm CMOS	22 nm FD-SOI	28 nm CMOS	180 nm CMOS	65 nm CMOS	65 nm CMOS	65 nm CMOS
I/Q technique	QVCO	QVCO	QVCO	QVCO	QVCO	QVCO	QVCO	QVCO	QVCO	RTWO	CRTWO	RTWO	RTWO	RTWO	Meta-RTWO
Frequency (GHz)	24	38	30.7	37.5	25.3	75	26	58.2	54	26.2	17.8	3.2	3.3	31.2	25.5
V_DD_ (V)	0.9	0.75	0.6	/	/	1	1	0.9	0.8	/	/	/	0.6	1	1
PN^c^1 MHz (dBc/Hz)	110.5	94.3	109.2	/	109	93^c^	99	93.5^c^	95.5	108.5	107^c^	129.8	129.5	99.4	112.7
Power (mW)	60	8.4	29.2	/	14.4	/	11.8	14	24	21	81^c^	/	15.16	22.7	17.4
FOM^c^1 MHz (dBc/Hz)	184	177	184.9	/	185.5	178^c^	176	177.4	187.6	183.6	172.9^c^	186.5	188	175.7	188.5
I/Q Phase error (°)	0.9^a^	0.8^a,b,c^	< 0.5^a,b^	1.8	1.8	4.2	0.36^a,b^	5	1.4	/	±1^a,c^	/	/	13.68	0.21

^a^Using phase adjustment structures

^b^Indirect measurement results (reverse calculation)

^c^Graphically estimated. / No relevant data in the literature

## Discussion

We proposed a Meta-RTWO based on spoof SPPs to solve the trade-off problem between low PN and small quadrature error in the conventional quadrature oscillators. Based on the proposed method, we can optimize both quadrature phase accuracy and FOM of the oscillators. A Meta-RTWO chip was fabricated via 65 nm CMOS technology. The measured results show that the Meta-RTWO chip is oscillated at 25.5 GHz with an approximate phase error of 0.21°, and the FOM value is 188.5 dBc/Hz. Compared with the similar literature published in recent years, the Meta-RTWO chip achieves better performance than those oscillators fabricated even by the 28 nm CMOS process in terms of the quadrature phase accuracy and FOM, even if it was fabricated by the backward 65 nm CMOS process, as shown in Table 1. In the future, the integration of Meta-RTWO with phase-locked loop (PLL) can further improve the performance. We believe that Meta-RTWO can be applied in various fields, including real-time calibration for 6 G massive MIMO antenna arrays, synchronization of terahertz-band quantum communication systems, and super-resolution beam forming techniques in multidimensional intelligent sensing frameworks.

## Materials and methods

### Simulation of dispersion curve

The dispersion curve of the spoof SPP TL is simulated using the Eigenmode Solver in the commercial software CST Microwave Studio. The boundary conditions along the propagation direction are set as periodic boundaries, and nonperiodic boundaries are set for electric or magnetic boundaries. The intrinsic phase shift between the periodic boundary conditions is then specified. The simulation is conducted for different phase shift conditions, and the resulting frequencies are used to obtain the dispersion curve.

### Impedance extraction

The impedance of the spoof SPP TL is extracted by performing a full-wave simulation using the Time Domain Solver in CST Microwave Studio. Firstly, a cross section of the spoof SPP TL with a quasi-TEM field distribution is selected to ensure that reflections are caused solely by impedance mismatch. A port with a known impedance is then placed at this cross-section. Using the reflection coefficient, the characteristic impedance at multiple frequency points is derived based on the relevant equations (see ref. ^46^). Finally, an interpolation method is applied to fit the characteristic impedance curve of the spoof SPP TL.

### Experimental testing

The chip is fixed on a Cascade probe station for testing. Its DC pads are connected to the power or ground on a PCB through gold wire bonding. High-frequency signals are output to the instruments for analysis via a GSGSG pad with a 150 μm pin pitch using a Z40-XD-GSGSG-150 probe and coaxial cables.

1. The RTWO spectrum and PN are tested using an R&S FSW43 spectrum analyzer. To obtain accurate signal output power, the losses of the probes and cables must be accounted for. The probe loss is obtained from the data sheet, whereas the cable loss is measured using an Agilent E8257D signal generator and an R&S FSW43 spectrum analyser. The probe and cable losses at frequencies of 25.5 GHz and 31.2 GHz are measured as 3.56 dB and 4.11 dB, respectively. These values, combined with the spectrum analyzer results, provide the true output power of the chip.
2. The phase difference of the RTWO output signal is measured by a VNA (KEYSIGHT PNA-X N5247A). The VNA receiver mode is used to measure the phase difference between the oscillator signals^17,47,48^. Traditional VNA calibration removes errors arising from cables and probes, bringing the measurement reference to the chip pad plane. To precisely measure the phase difference between the oscillating signals, an additional internal receiver synchronization calibration is performed based on the traditional calibration method.

## Supplementary information

SI-A plasmonic meta-rotary traveling-wave oscillator with ultrahigh phase accuracy and figure of merit

## Data Availability

The data that support the plots within this paper and other findings of this study are available from the corresponding author upon reasonable request.
