# Supplementary material for: A plasmonic meta-rotary travelling-wave oscillator with ultrahigh phase accuracy and figure of merit
Source: Light Sci Appl. 2025 Aug 21;14:284. doi: 10.1038/s41377-025-01966-z (PMC12370945; doi:10.1038/s41377-025-01966-z)
Supplement: Supplementary file 1 — SI-A plasmonic meta-rotary traveling-wave oscillator with ultrahigh phase accuracy and figure of merit [file 41377_2025_1966_MOESM1_ESM.docx]

**Supplementary information for**

**A plasmonic meta-rotary travelling-wave oscillator with ultrahigh phase accuracy and figure of merit**

Da Yue Yao^1,2,3^, Hao Chi Zhang^1,2,3🖂^, Pei Hang He^1,2,3^, Jia Jie Shen^1,2,3^, Jia Wen Zhu^1,2^, Peigen Zhou^1^, Xin Yu Zhang^1,2^, Le Peng Zhang^1,2^, Li Jie Wu^1,2^, Cun Yue Wei^1,2^, Rui Wen Shao^1,2^, Yi Fan^1,2^, Yang Zhao^1,2^, Jixin Chen^1^, Wei Hong^1^ and Tie Jun Cui^1,2🖂^

1 State Key Laboratory of Millimeter Waves, Southeast University, Nanjing, China.

2 Institute of Electromagnetic Space, Southeast University, Nanjing, China.

3 These authors contributed equally: Da Yue Yao, Hao Chi Zhang, Pei Hang He, Jia Jie Shen and Jia Wen Zhu.

E-mails: hczhang0118@seu.edu.cn; tjcui@seu.edu.cn

**This file includes:**

Supplementary Information 1: Introduction to other metamaterial transmission lines and slow-wave structures

Supplementary Information 2: Parasitic effects of -*Gm* cells on wave propagation

Supplementary Information 3: Derivation of Meta-RTWO startup conditions

Supplementary Information 4: Analysis of RTWO compared to multi-core SWOs

Supplementary Information 5: The process details of 65nmCMOS

Supplementary Information 6: The dispersion analysis of spoof SPPs

Supplementary Information 7: Analysis of the loss in spoof SPP TL and the efficiency of Meta-RTWO

Supplementary Information 8: Design approach for Meta-RTWO resonator

Supplementary Information 9: RTWO circuit structure

Supplementary Information 10: Analysis of RTWO startup time

Supplementary Information 11: Measurement method of signal phase difference

Supplementary Figures S1-S15

Supplementary Equations (S1)-(S12)

**Supplementary Information 1: Introduction to other metamaterial transmission lines and slow-wave structures**

Other commonly used transmission-line metamaterials include left-handed materials (LHMs) ^S1^ and defected ground structures (DGSs)^S2^. LHMs are a class of metamaterials characterized with simultaneously negative permittivity and permeability, as illustrated in Figure S1a. A distinctive feature of LHMs is that, upon electromagnetic wave incidence at the interface between an LHM and a conventional dielectric, the refracted wave appears on the same side of the interface normal as the incident wave. However, the transmission lines based on LHMs generally suffer from drawbacks such as narrow bandwidth, high losses, and structural non-uniformity, making them unsuitable for RTWO applications. Defected ground structure (DGS), on the other hand, is a technique that introduces periodic or aperiodic patterns into the ground plane of a microstrip line to achieve specific electromagnetic characteristics, as shown in Figure S1b. Despite its advantages, DGS structures are typically complex and challenging to implement in the standard CMOS fabrication processes.


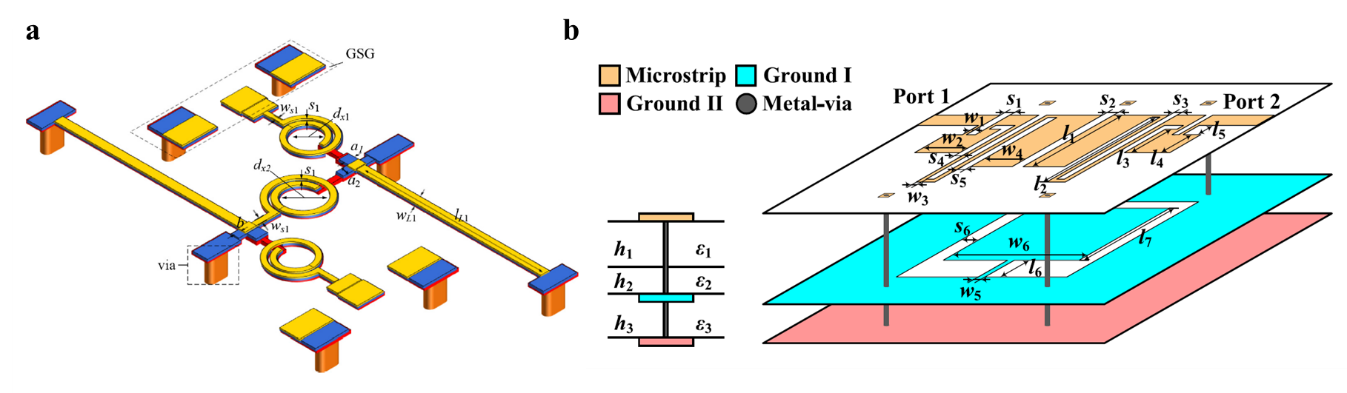


**Figure S1: Other transmission-line metamaterials.** a, Structural diagram of a left-handed material (LHM) transmission line ^S1^. b, Structural diagram of a (DGS) transmission line ^S2^.

Common slow-wave structures include staggered double vane structure^25^, ladder line structure ^26^, and folded waveguide structure^27,S3-S5^. However, these structures are not compatible with planar fabrication processes. As an alternative, serpentine traces compatible with planar processes are widely employed as slow-wave transmission lines, though they exhibit high loss and limited tuning flexibility. Figure S2 presents the simulation results of transmission losses (magnitude/phase of S_21_) for the spoof SPP and serpentine transmission lines under the condition of equal physical and electrical lengths. From the figure, it can be observed that in the range of 20-30 GHz, the loss of spoof SPP is consistently lower than that of the serpentine transmission line. At 25.5 GHz, the loss for spoof SPP is 0.008 dB/degree, whereas for the serpentine transmission line, it is 0.013 dB/degree. Therefore, the choice of spoof SPP, which offers low loss, high design flexibility, and compatibility with planar processes, is made to realize the Meta-RTWO.


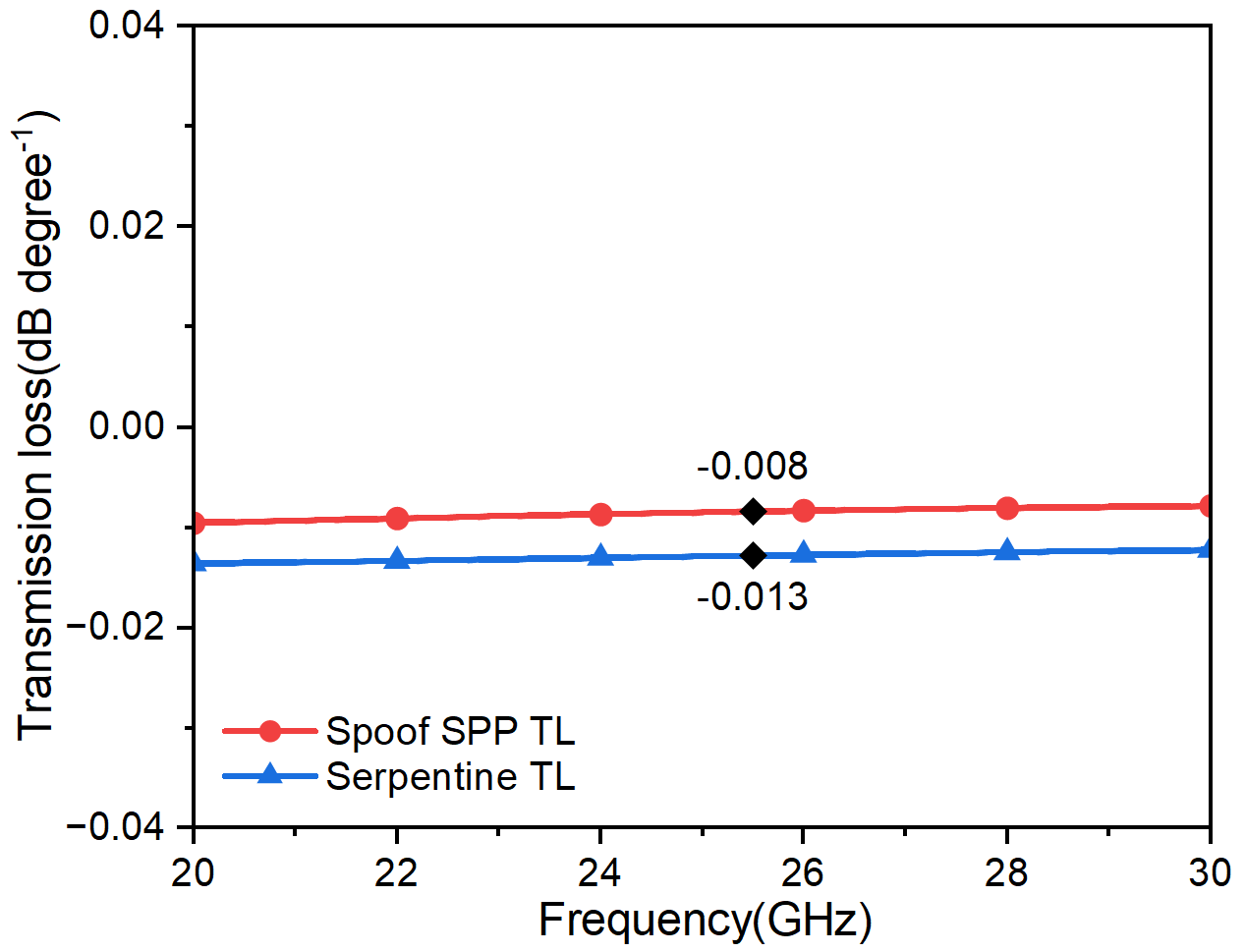


**Figure S2: Simulation results of transmission loss of Spoof SPP and serpentine**

**Supplementary Information 2: Parasitic effects of -*Gm* cells on wave propagation**

RTWO is composed of a differential transmission line (TL) with the crossed head and tail configurations, along with an uniformly distributed -*Gm* cells, as illustrated in Figure S3. The primary parasitic effect of the -*Gm* cells is parasitic capacitance *C_i_* ^S6^, which introduces phase shift capability. Consequently, the phase shift (π/4) between nodes *P_i_* and *P*[(*_i_*_+1)mod8]_ results from both the TLs and the parasitic capacitance of the -*Gm* cells, as expressed in Eq. (S1).

(S1)


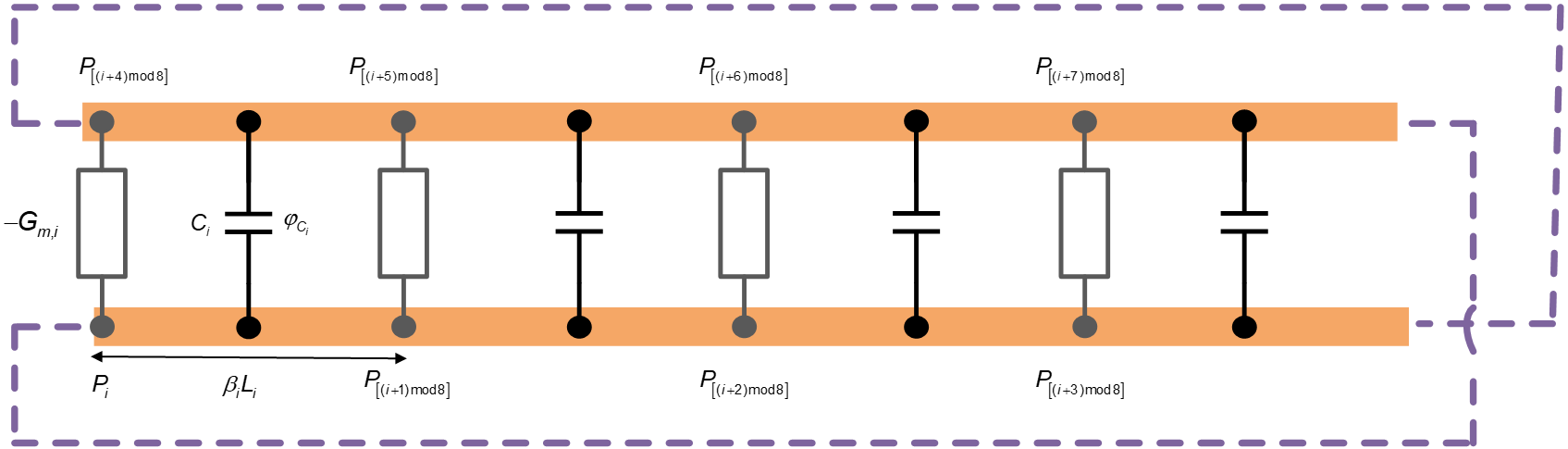


**Figure S3: Equivalent model of RTWO considering parasitic effects of -*Gm* cells.**

In other words, due to the parasitic capacitance of the -*Gm* cells, the phase shifts (*β_i_L_i_*) of each TLs are typically less than π/4 at the RTWO oscillation frequency (as shown in Fig. 4 of the main text). However, since we employ identical -*Gm* cells in the design, maintaining equal phase shifts (*φ_Ci_*) produced by each cell, this paper needs only focus on optimizing instances where (*β_i_L_i_*) is unequal due to the asymmetry of the TLs in each segment.

**Supplementary information 3: Derivation of Meta-RTWO startup conditions**

To examine the transfer function of Meta-RTWO, we conduct a decoupling operation on the RTWO model, as shown in Fig. 2b. RTWO depicted in Fig. 2a can be conceptualized as a distributed oscillator composed of four *-Gm* cells. When observed from the terminals of any -*Gm* cell, RTWO can be considered as a network of the input *V*_in_ and output *V*_out_ of a fully differential distributed amplifier^1,2^ connected together. Assuming that the reflections between segments are negligible and the voltages across the negative resistance units have equal amplitudes with phase difference of 180°, the discrepancy in voltages between the terminals of the negative resistance signal is a factor of e*^j^*^π^. For any two intermediate stages, *V_i_* and *V*[(*_i_*_+1)mod8]_, the following transfer function is valid:

$H(\omega)_{\left[(i+1)\mathrm{mod}8\right]}=\frac{V_{\left[(i+1)\mathrm{mod}8\right]}}{V_{i}}=(1-\mathrm{G}_{m}\mathrm{Z}_{0})\text{e}^{-\gamma_{i}L_{i}}$ (S2)

where (*i*+1)mod 8 denotes the remainder of the division by 8, Gm is the transconductance of the -*Gm* cell, Z_0_ represents the characteristic impedance of TL, and *γ* is the propagation constant of TL. According to microwave theory, *γ*=*α*+j*β*, in which *α* is the attenuation constant to characterize the reduction in the wave amplitude along TL, and *β* is the phase constant to indicate the phase shift of the wave as it propagates along TL. The transfer function of RTWO shown in Fig. 2b can be expressed by

$H(\omega)=\frac{V_{out}}{V_{in}}=\prod_{j=1}^{4} H(\omega)_{\left[(i+j)\mathrm{mod}8\right]}*\frac{V_{i}}{V_{in}}=-(1-\mathrm{G}_{m}\mathrm{Z}_{0})^{4}\text{e}^{-A}$ (S3)

in which $A=\gamma(\omega)_{i}L_{i}+\sum_{j=1}^{3} \gamma(\omega)_{\left[\left(i+j\right)\mathrm{mod}8\right]}L_{\left[\left(i+j\right)\mathrm{mod}8\right]}$. Substituting Eq. (S3) into the oscillation conditions gives the following conditions to be satisfied for the RTWO oscillation:

$\left\{\begin{matrix} (1-\mathrm{G}_{m}\mathrm{Z}_{0})^{4}\text{e}^{-\alpha(\omega)L}\geq1 \\ \text{e}^{\text{-j}B}=-1\Rightarrow B=\pi\end{matrix}\right.$ (S4)

where$B=\beta(\omega)_{i}L_{i}+\sum_{j=1}^{3} \beta(\omega)_{\left[\left(i+j\right)\mathrm{mod}8\right]}L_{\left[\left(i+j\right)\mathrm{mod}8\right]}$. In general, the amplitude balance condition can be satisfied by designing the size of the MOS -*Gm* cells and changing the Gm value. From Eq. (S4), the phase balance condition required for oscillation can be further deduced as

$\left\{\begin{matrix} \sum_{i=1}^{8} \beta(\omega)_{i}L_{i}=2\pi\\ \beta(\omega)_{i}L_{i}=\beta(\omega)_{i+4}L_{i+4},i=1,2,3,4 \end{matrix}\right.$ (S5)

Hence the RTWO oscillation requires that the total electrical length of the resonator is equal to 2π and, and the electrical lengths of inner and outer loop TLs connecting any negative resistance unit are equal.

**Supplementary Information 4: Analysis of RTWO compared to multi-core SWOs**

Takinami *et al.* demonstrated that RTWO can be modeled as a superposition of multiple standing-wave oscillators (SWOs^S7^) that have been injection locked^S8^, as illustrated in Figure S4. The complete dynamic equations for the four coupled SWOs, derived from the generalized Adler’s equation^S7^, are presented below:

$\frac{d\theta_{1}}{dt}=\omega_{0}+\frac{\omega_{0}}{2Q}\times\frac{\sin\left(\beta_{1}L_{1}\right)+\sin\left(-\beta_{8}L_{8}\right)}{\eta+\cos\left(\beta_{1}L_{1}\right)+\cos\left(-\beta_{8}L_{8}\right)}$ (S6)

$\frac{d\theta_{2}}{dt}=\omega_{0}+\frac{\omega_{0}}{2Q}\times\frac{\sin\left(\beta_{2}L_{2}\right)+\sin\left(-\beta_{1}L_{1}\right)}{\eta+\cos\left(\beta_{2}L_{2}\right)+\cos\left(-\beta_{1}L_{1}\right)}$ (S7)

$\frac{d\theta_{3}}{dt}=\omega_{0}+\frac{\omega_{0}}{2Q}\times\frac{\sin\left(\beta_{3}L_{3}\right)+\sin\left(-\beta_{2}L_{2}\right)}{\eta+\cos\left(\beta_{3}L_{3}\right)+\cos\left(-\beta_{2}L_{2}\right)}$ (S8)

$\frac{d\theta_{4}}{dt}=\omega_{0}+\frac{\omega_{0}}{2Q}\times\frac{\sin\left(\beta_{4}L_{4}\right)+\sin\left(-\beta_{3}L_{3}\right)}{\eta+\cos\left(\beta_{4}L_{4}\right)+\cos\left(-\beta_{3}L_{3}\right)}$ (S9)

where *θ_i_* represents the phase of the wave at the *P_i_* node, *η* denotes the scaling factor (if a sinusoidal voltage distribution along the resonator, *η* is about 1.414), and Q refers to the quality factor. Assuming that the four SWOs are injection-locked at the same oscillation frequency *ω*_osc_, we have *dθ_i_*/*dt*=*ω*_osc_ and *ω*_osc_=*ω*_0_. This leads to two potential solutions for the aforementioned equation:

$\beta_{i}(\omega_{0})L_{i}=\frac{\pi}{4},i=1,2,\cdots8$ (S10)

$\beta_{i}(\omega_{0})L_{i}=0$ (S11)

It was demonstrated that Eq. (S11) does not yield stable results in Ref. 2. Consequently, the RTWO can operate stably only if the condition specified in Eq. (S10) is met, meaning that the electrical lengths of each segment of the TL must be equal π/4 at the same frequency.


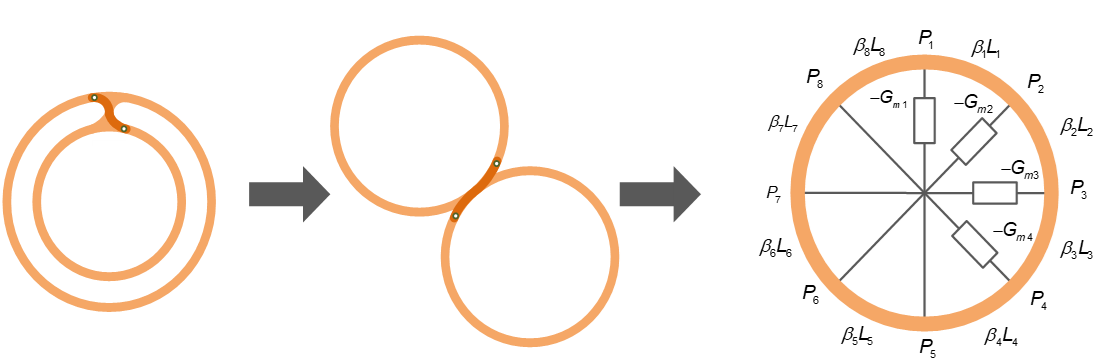


**Figure S4: Analysis of RTWO compared to multi-core SWOs.**

**Supplementary Information 5：The process details of 65nmCMOS**

The CMOS process production can be divided into the active region fabrication and the metal interconnect fabrication. The former is mainly to complete the production of MOS transistors, while the latter is mainly to complete the production of contact holes and electrical metal connections. The following are the detailed process steps.

**1) Active region fabrication**

**Selection of P-type substrate.** A heavily doped P^+^-type silicon wafer with a specific crystal orientation is selected as the starting material. A lightly doped P^-^-type epitaxial layer is then grown on its surface via a vapor-phase epitaxy process, as illustrated in Figure S5a.

**N-well formation.** After defining the N-well regions using photolithography, N-type dopants such as phosphorus or arsenic are implanted using an ion implanter. A high-temperature annealing process is subsequently carried out to activate the dopants and repair lattice damage, thereby forming the N-well region, as shown in Figure S5b.

**P-well formation.** A second photolithography step is used to define the P-well regions, followed by the implantation of P-type dopants such as boron. Rapid thermal annealing is employed to activate the dopants and precisely control lateral diffusion, ensuring sufficient spacing from adjacent N-wells to prevent latch-up effects, as depicted in Figure S5c.

**Polysilicon gate deposition.** A high-quality gate oxide layer is thermally grown, followed by the deposition of phosphorus-doped polysilicon using Low Pressure Chemical Vapor Deposition (LPCVD). Dry etching is then used to pattern submicron gate structures, as shown in Figure S5d.

**NMOS source/drain implantation.** After sidewall spacer formation, arsenic or phosphorus ions are implanted into the NMOS regions. A spike annealing process is applied to activate the dopants and form shallow junctions, effectively suppressing short-channel effects, as illustrated in Figure S5e.

**PMOS source/drain implantation.** Boron or BF_2_ ions (a compound of boron used to enhance doping concentration in PMOS source/drain regions) are implanted into the PMOS regions. A subsequent nickel silicide annealing process is conducted to form low-resistance contacts, significantly reducing the contact resistance of the source and drain regions, as shown in Figure S5f.


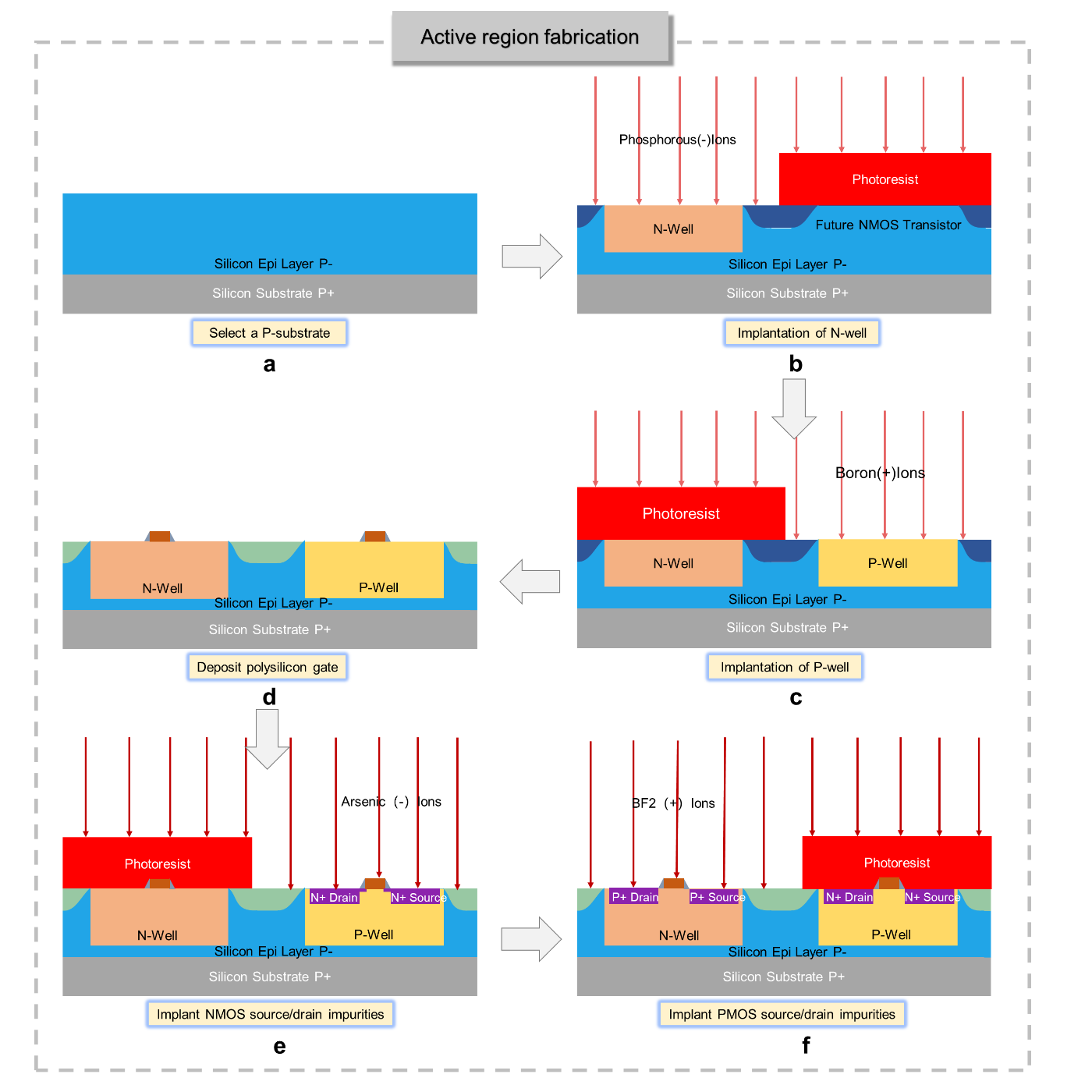


**Figure S5: Active region fabrication.** a, Selection of P-type substrate. b, N-well formation. c, P-well formation. d, Polysilicon gate deposition. e, NMOS source/drain implantation. f, PMOS source/drain implantation.


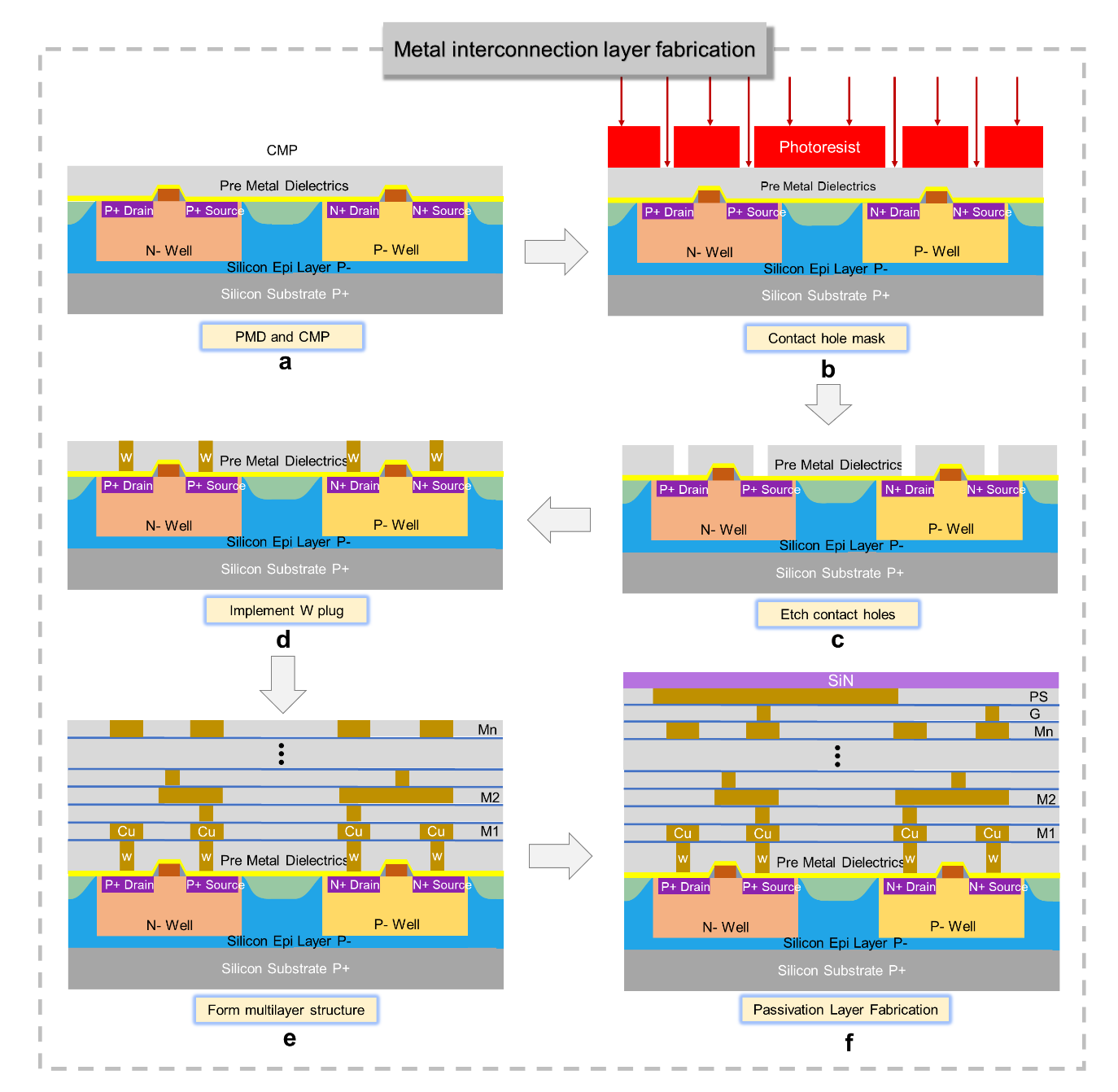


**Figure S6: Metal interconnect fabrication.** a, Planarization of the isolation layer. b, Etch pattern formation. c, Contact via formation. d, Via plug formation. e, Multilayer structure formation. f, Passivation Layer Fabrication.

**2) Metal interconnect fabrication**

**Planarization of the isolation layer.** An etch-stop layer and a pre-metal dielectric (PMD) isolation layer are deposited using Plasma Enhanced Chemical Vapor Deposition (PECVD). Chemical-Mechanical Polishing (CMP) is then applied to achieve surface planarization, compensating for topographical variations caused by the underlying gate structures, as illustrated in Figure S6a.

**Etch pattern formation.** Photolithography is used to define the contact via pattern. These vias must connect precisely to the fine-featured source/drain terminals and gates, with minimal interconnect spacing to maximize layout density, as shown in Figure S6b.

**Contact via formation**. The PMD layer is etched using the patterned photoresist as a mask to open the contact vias. Unwanted photoresist is subsequently removed via ashing, as depicted in Figure S6c.

**Via plug formation**. A titanium nitride (TiN) or titanium (Ti) adhesion layer is deposited within the contact vias, followed by the deposition of a tungsten (W) layer. The adhesion layer ensures robust bonding between the PMD and the tungsten film. CMP is then performed to remove the excess W and adhesion layers above the PMD surface, thereby forming tungsten plugs (W-plugs), as illustrated in Figure S6d.

**Multilayer structure formation.** Steps 1 through 4 are repeated to form multiple layers of metal interconnects, enabling complex circuit routing in advanced integrated circuits, as shown in Figure S6e.

**Passivation layer fabrication**. To protect the circuit from environmental contaminants, mechanical damage, and moisture, a passivation layer is deposited. This typically consists of a combination of phosphosilicate glass (PSG) and silicon nitride (SiN), which effectively blocks moisture and mobile ion penetration from the external environment, as depicted in Figure S6f.

**Supplementary Information 6: The dispersion analysis of spoof SPPs**

The conventional spoof SPP structure integrates a central stripline with periodically arranged open-circuited stubs, as illustrated in Figure S7. The key structural parameters include the unit cell period *p_1_*, the main strip width s1, the cutoff stub length w1, and the cutoff stub width *a_1_*. The dispersion curve intuitively demonstrates the capability of spoof SPPs to modulate the phase constant of waves. And the farther the dispersion curve deviates from the light line, the larger the phase constant. The dispersion curves of the traditional spoof SPP units are simulated using the Eigenmode Solver of commercial software CST Microwave Studio. The dispersion curves of the conventional spoof SPP structures under different values of *p_1_*, *s_1_*, *w_1_*, and *a_1_* are presented in Figure S8. It can be observed that variations in *s_1_*, *a_1_*, and *p_1_* have a negligible effect on the phase constant of the spoof SPP. In contrast, altering *w_1_* leads to a significant change in the phase constant. However, achieving the desired phase constant in conventional spoof SPP structures typically requires a large *w_1_*, which results in an excessively large structure and substantial chip area consumption.

**
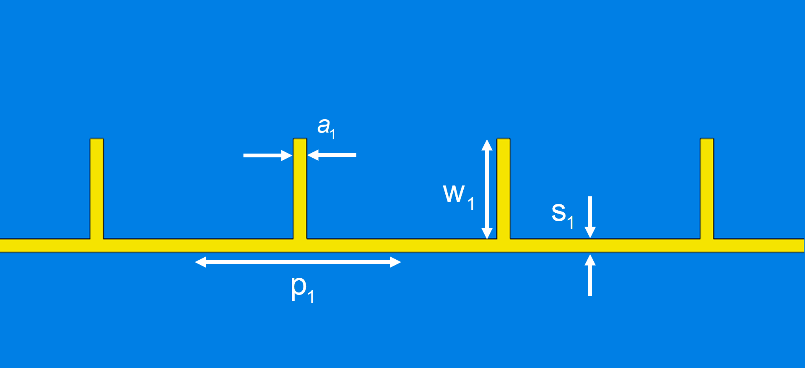
**

**Figure S7: The conventional spoof SPP unit.**

**
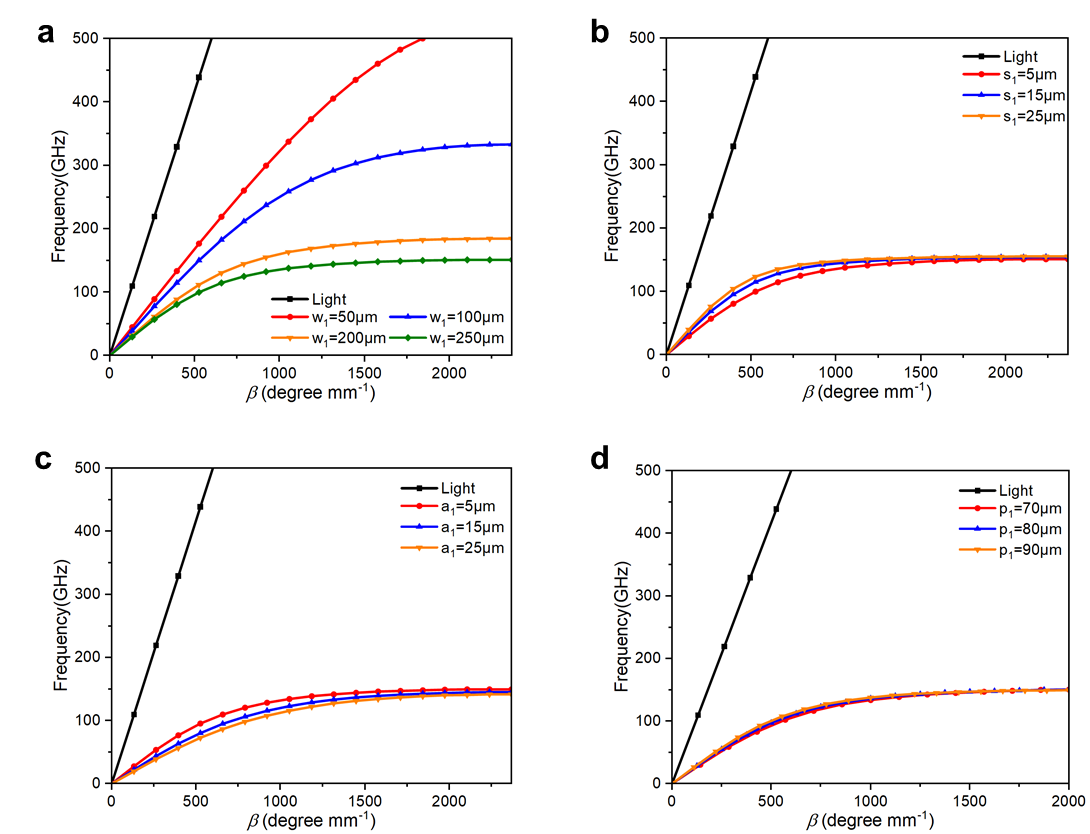
**

**Figure S8: The dispersion curves of the conventional spoof SPP units.** a, Dispersion curves of conventional spoof SPP units for different values of w, where *p*=76 μm, *s*=5 μm, and *a*=5 μm. b, Dispersion curves of conventional spoof SPP units for different values of s, where *p*=76 μm, *a*=5 μm, and *w*=250 μm. c, Dispersion curves of conventional spoof SPP units with different a, where *s*=3 μm, *w*=250 μm, and *p*=76 μm. d, Dispersion curves of conventional spoof SPP units with different p, where *a*=5 μm, *s*=5 μm, and *w*=250 μm.

To address this limitation, we propose a miniaturized spoof SPP structure, as illustrated in Figure S9. This design features a primary transmission strip loaded with periodically bent open-circuit stubs placed adjacent to its sides. The core idea behind the miniaturized design is to transform the straight short-circuit stubs of the conventional spoof SPP structure into meandered configuration. This approach effectively increases the equivalent length of *w_1_* without enlarging the physical footprint. A more detailed schematic of the miniaturized spoof SPP cell and its structural parameters is provided in Figure S9. It consists of a primary transmission strip flanked by periodically meandered open-circuit stubs. In this structure, p denotes the period length, s is the width of the main transmission strip, w is the radial length of the short-circuit stub, a represents both the width and spacing of the stub, and *N* is the order of the meander. The dispersion characteristics of the miniaturized spoof SPP under varying parameters are shown in Figure S10. From the plots, it can be observed that the parameter *p* has minimal influence on the phase constant. Conversely, the phase constant decreases as the width *s* of the metal strip increases. Considering process limitations, the minimum feasible linewidth *s*=2  μm is selected for fabrication. Furthermore, the phase constant increases with larger values of *a*, *N*, and *w*. Among these, the parameter *a* exhibits only a slight impact on the phase response. Therefore, to enhance miniaturization, *a* is fixed at 2  μm. On the other hand, both *N* and *w* significantly influence the phase constant. In this design, *N* is utilized for coarse adjustment over a broad range, while *w* serves as a fine-tuning parameter. Given that the phase constant requirements for different segments of the RTWO resonator do not vary significantly, the meander order is fixed at *N*=4, and precise phase control is achieved by adjusting the radial length *w* of the short-circuit stubs.


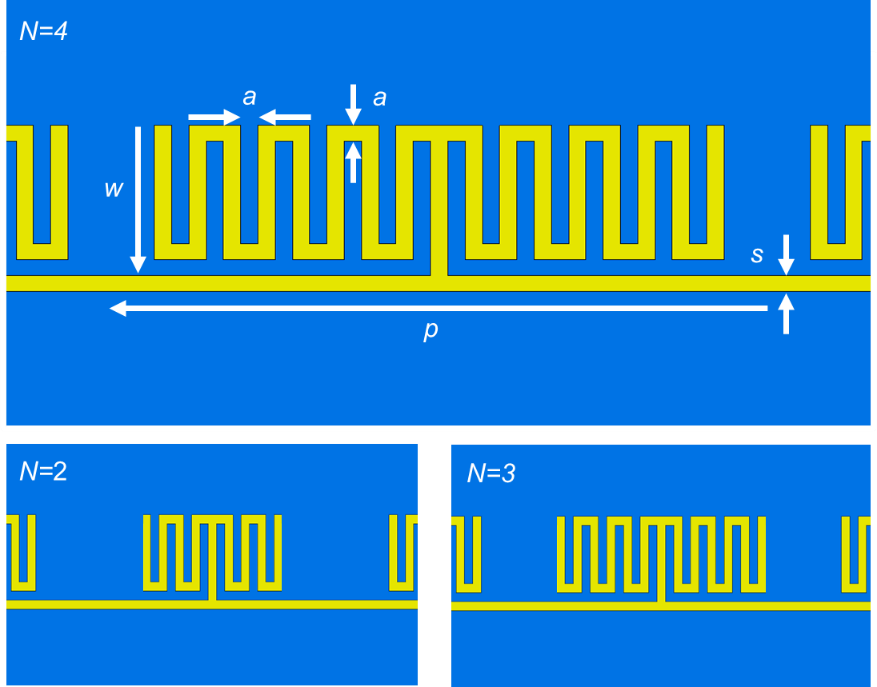


**Figure S9: The miniaturized spoof SPP unit.**


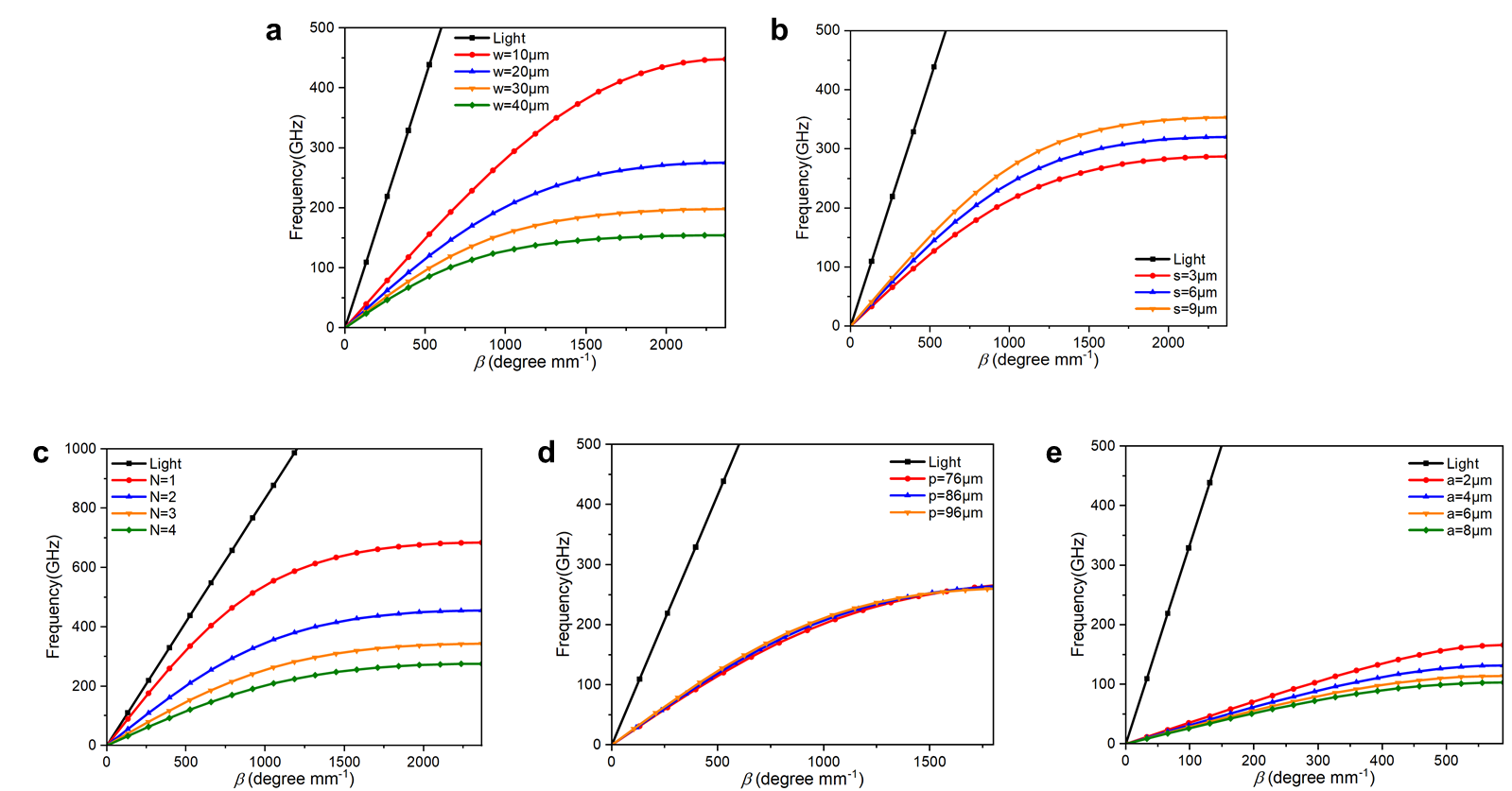


**Figure S10: The dispersion curves of the miniaturized spoof SPP units.** a, Dispersion curves of spoof SPP units for different values of *w*, where *p*=76 μm, *w*=20 μm, and *N*=4. b, Dispersion curves of spoof SPP units for different values of *s*, where *p*=76 μm, *s*=2 μm, and *N*=4. c, Dispersion curves of spoof SPP units with different *N*, where *s*=2 μm, *w*=20 μm, and *p*=76 μm. d, Dispersion curves of spoof SPP units with different *p*, where *a*=2 μm, *s*=2 μm, and *w*=20 μm. e, Dispersion curves of spoof SPP units with different *a*, where *s*=2 μm, *w*=20 μm, and *p*=306 μm.

**Supplementary Information 7: Analysis of the loss in spoof SPP TL and the efficiency of Meta-RTWO**

The transmission loss of the spoof SPP TL can be characterised using the ratio of the transmission coefficient S_21_ magnitude and phase. The efficiency of the oscillator is generally expressed as the ratio of the power of the DC and output AC signals, as shown in Eq. S12.

**1) The loss of spoof SPP TL**

The simulated transmission loss results for the unilateral non-intersecting structure of the Meta-RTWO resonator (spoof SPP TL, *L*_1_) and the corresponding MS-RTWO (MS TL, *L*_1_) are shown in Figure S11. The simulation results indicate that both transmission lines exhibit losses below 0.009 dB/degree in the 20-30 GHz range. At 25.5 GHz, the MS TL loss is 0.006 dB/degree, which is 0.002 dB/degree higher than the spoof SPP TL's 0.008 dB/degree. And thus the loss brought by the introduction of the spoof SPP TL is almost negligible.


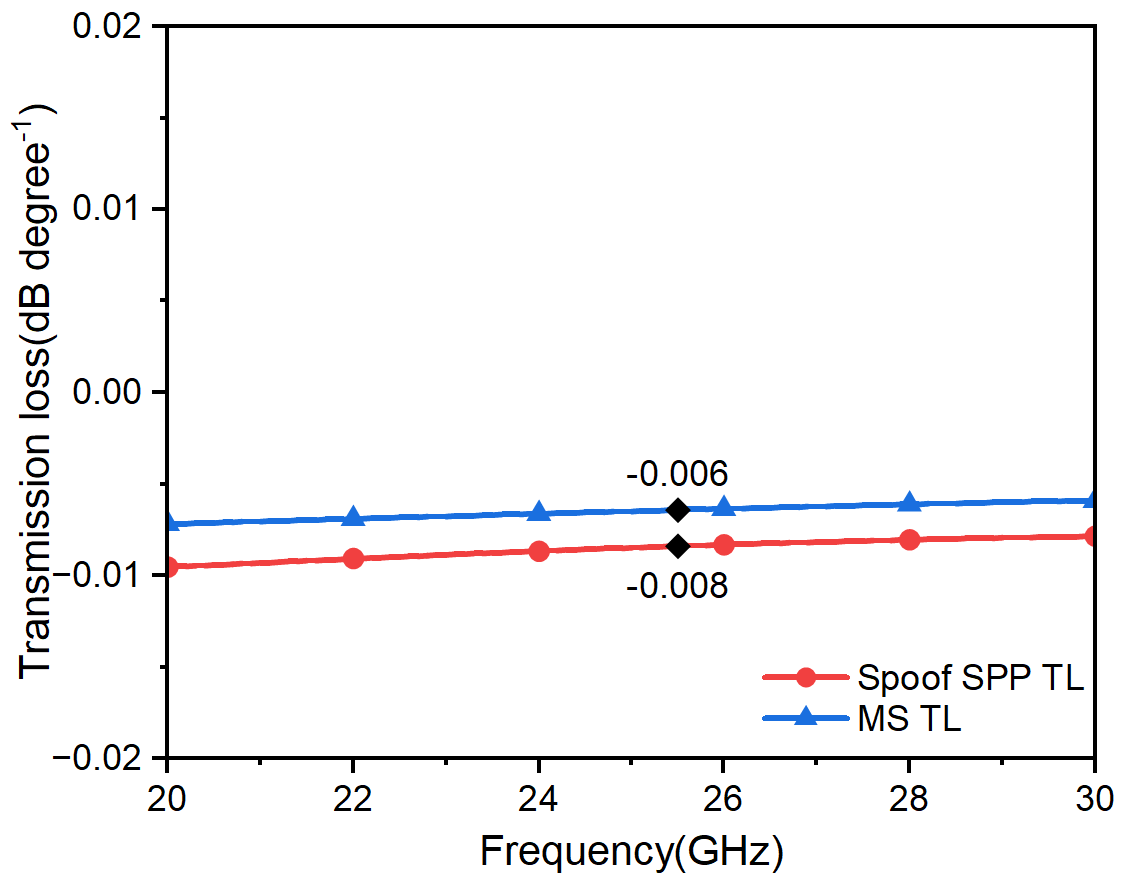


**Figure S11: The transmission loss simulation result of spoof SPP and MS TLs.**

**2) The efficiency of oscillator**

For the efficiency of the oscillator, the Meta-RTWO has a DC power consumption of 17.4 mW and an output power of 2.2 dBm, and the efficiency is 9.5% according to the following formula. The MS-RTWO has a DC power consumption of 22.7 mW and an output power of 1.91 dBm, and its efficiency is 6.8% according to the following equation:

$\eta=\frac{P_{out}}{P_{\text{dc}}}$ (S12)

It can be seen that the introduction of the spoof spp TL brings about an improvement in the efficiency of Meta-RTWO. This is attributed to the strong dispersion modulation capability of the spoof SPP transmission lines, which enables the energy supplied by each negative resistance unit in the Meta-RTWO resonator to be effectively superimposed.

**Supplementary Information 8: Design approach for Meta-RTWO resonator**

According to the topology illustrated in Fig. 2(a), the Meta-RTWO resonator comprises four sequentially connected transmission line segments. Among them, three segments adopt the uncrossed spoof SPP configuration, as shown in Fig. 4(a), while the remaining segment employs a one-sided crossed jumpered spoof SPP structure, as shown in Fig. 4(b). The design principle of the spoof SPP-based Meta-RTWO resonator lies in tailoring the phase constants of each TL segment to compensate for the slight physical length discrepancies among the segments, thereby ensuring that all segments exhibit equal electrical lengths. As indicated in the topology diagram, the differences in physical length between the segments are relatively minor. Therefore, fine-tuning of the phase constants is achieved by adjusting the parameter w. To begin with, the phase constants of the inner ring uncrossed spoof SPP transmission lines (*L*_1or2or8_) are extracted using the Eigenmode Solver in CST Microwave Studio to accurately reproduce the dispersion characteristics, as shown in Figure S12. Since the outer ring segments (*L*_5or6or4_) are physically longer than those in the inner ring, their phase constants must be smaller to match the electrical lengths. This implies that the w values of the spoof SPP units in the outer ring must be reduced accordingly. Since the two wires of the crossover structure span the inner and outer loops, the w-values of the four spoof SPP cell structures corresponding to the two wires have to be designed individually to satisfy *β*_3_*L*_3_=*β*_7_*L*_7_=*β_i_L_i_*. Finally, the scattering parameters of the complete transmission line structure are simulated using the time-domain solver in CST Microwave Studio. The design is considered successful when the phase shifts across all segments are nearly identical. In this design, the maximum phase deviation among segments is only 0.16°, as shown in Fig. 4(c).


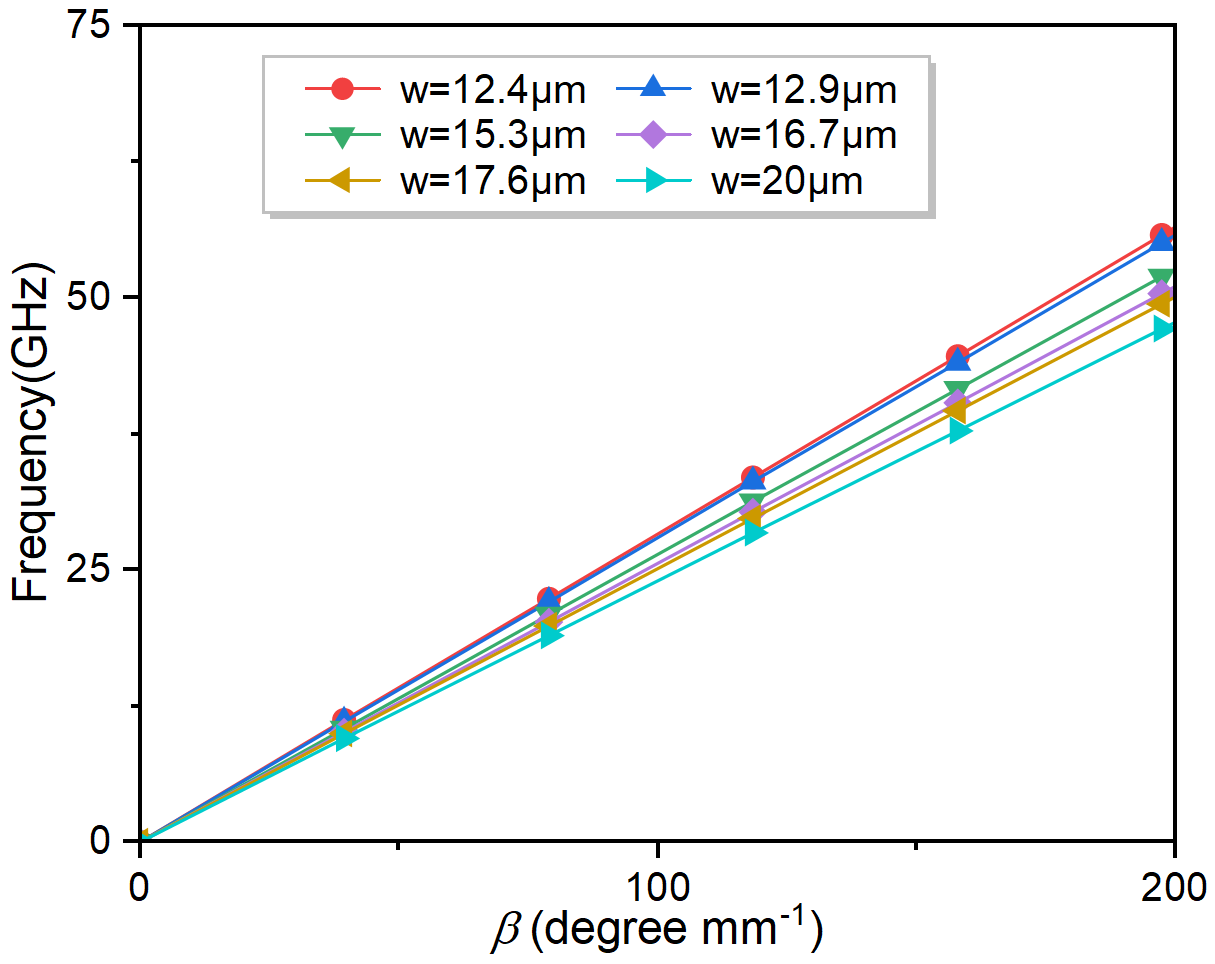


**Figure S12: Dispersion curves of spoof SPP for each segment of Meta-RTWO resonator.**

**Supplementary information 9: RTWO circuit structure**

The RTWO circuit architecture is shown in Figure S13. SN_n_ and SN_p_ form a pair of differential signals, while SN_1_ and SN_2_, SP_1_ and SP_2_, respectively, form quadrature signals. In the experiment, without loss of generality, SN_1_ and SN_2_ are selected as the test subjects. In the design, a common-source structure with differential inductors as the load is chosen as the Buffer. This Buffer amplifies the oscillation signals and isolates the core circuit from the load. A constant gm bias circuit is designed to provide the buffer with a stable bias voltage. The bias circuit is illustrated in Figure S14. The current sources PM_1_, PM_2_, NM_3_, NM_4_, and resistor R_2_ form a "Constant gm" structure, which generates a positive temperature coefficient current unaffected by power supply voltage variations. This current compensates for the reduction in gm caused by high temperatures in the MOS transistors. The size of NM_6_ matches that of NM_3_, replicating its 20 μA current. PM_4_ and PM_5_ are sized five times that of PM_3_, resulting in a current of 100 μA.


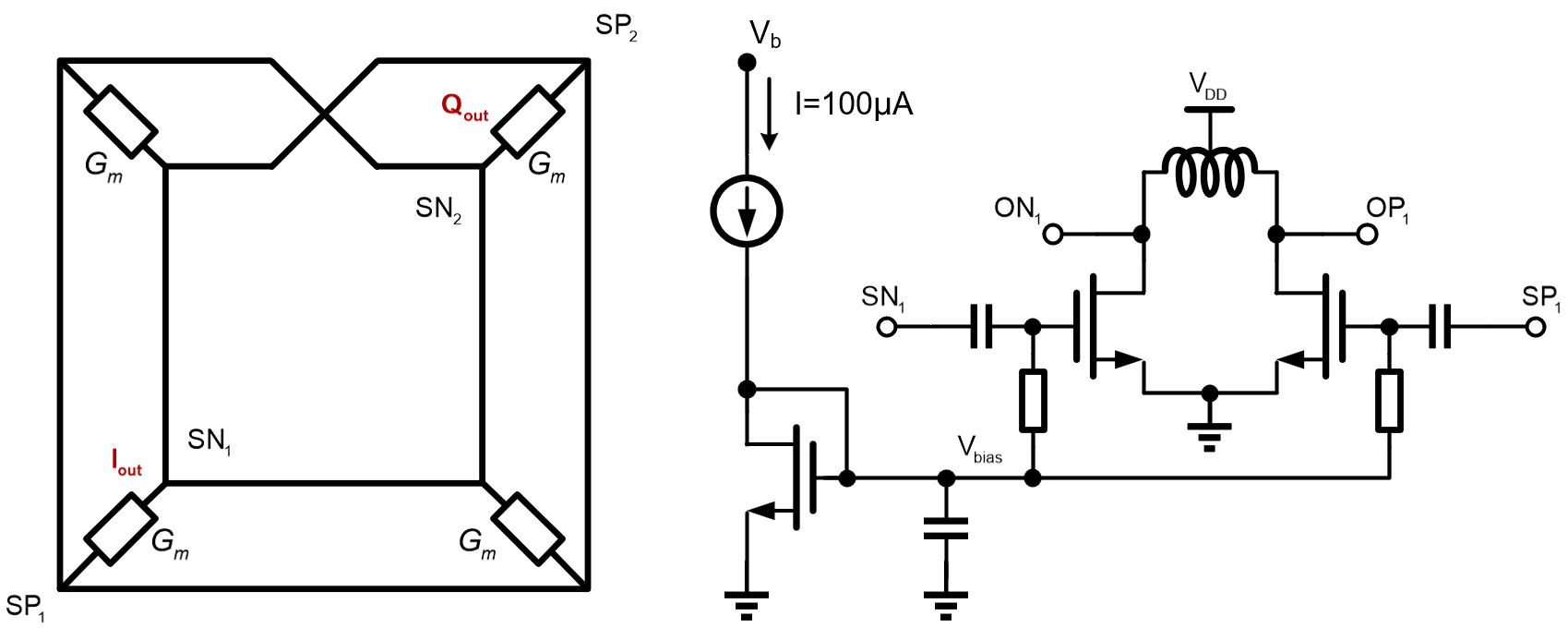


**Figure S13: The architecture diagram of the RTWO circuit.**


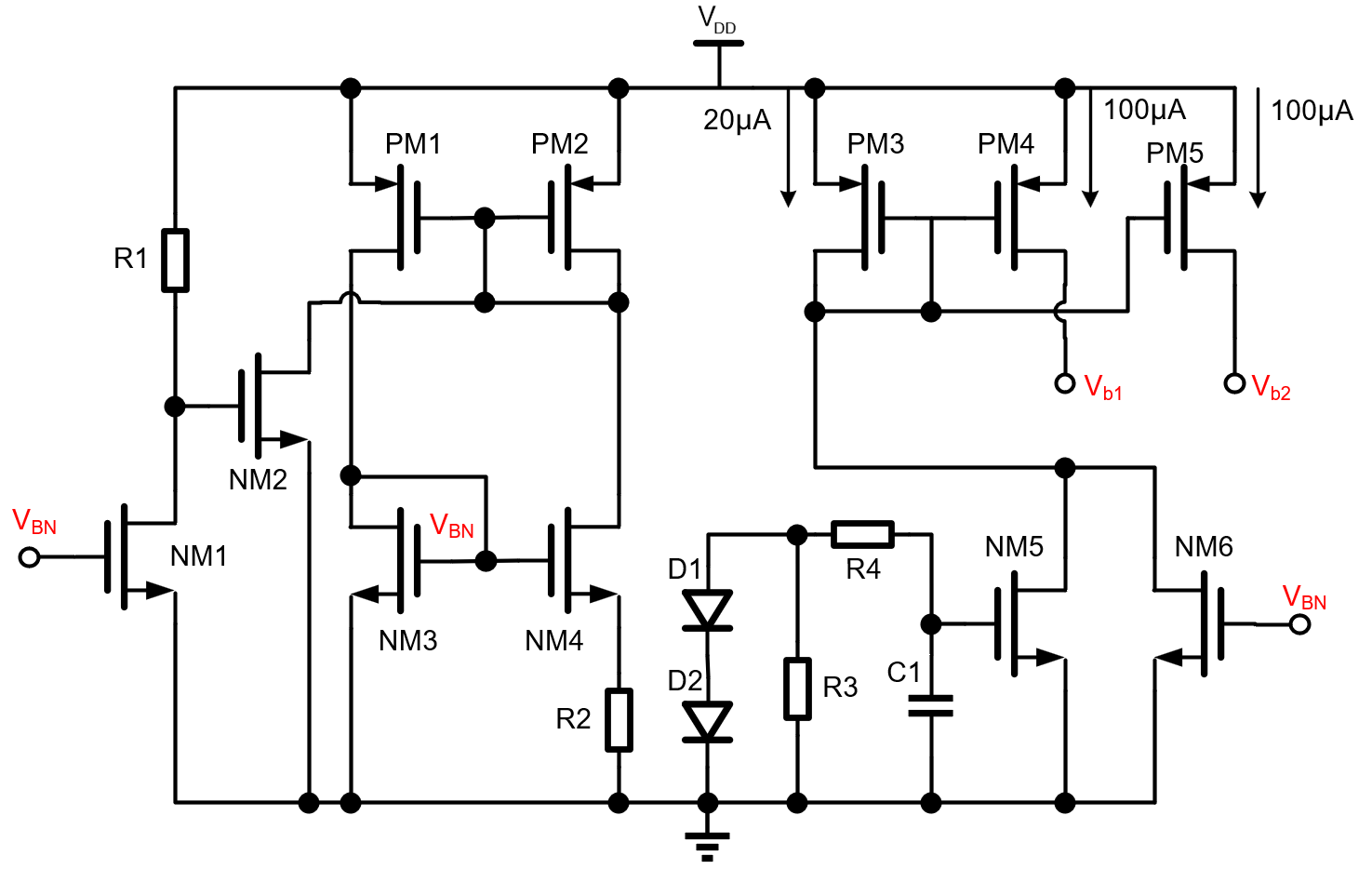


**Figure S14: Bias circuit diagram.**

**Supplementary Information 10: Analysis of RTWO startup time**

The simulations of oscillator startup transients (Figure S15) reveal comparable stabilization characteristics between the implementations of Meta-RTWO and MS-RTWO. Meta-RTWO achieves stabilization at 0.358 ns post-activation (9 oscillation cycles), whereas MS-RTWO stabilizes at 0.295 ns (9 cycles). These results demonstrate that the spoof SPP integration introduces negligible temporal overhead to the startup process.


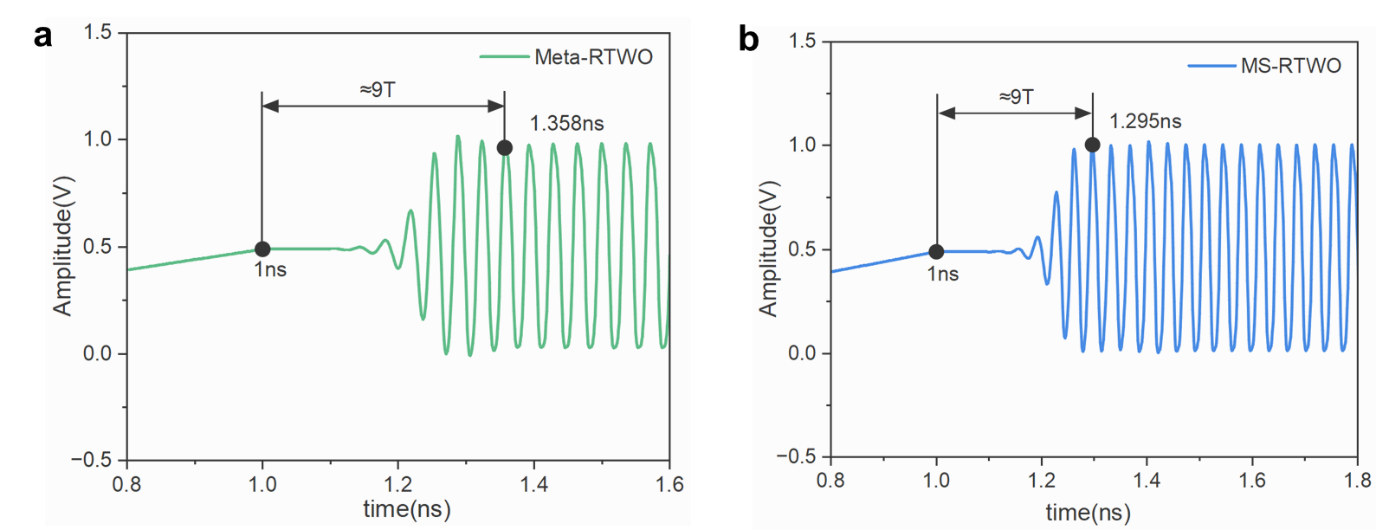


**Figure S15: Startup time simulation of RTWO.** a, Startup time simulation of Meta-RTWO. b, Startup time simulation of MS-RTWO.

**Supplementary Information 11: Measurement method of signal phase difference**

Commonly used methods for measuring phase accuracy include the direct measurement using an oscilloscope, and indirect measurement using image rejection ratio and direct measurement using a Vector Network Analyzer (VNA).

**1) Direct measurement using an oscilloscope**

Phase accuracy can be obtained by directly measuring the time-domain waveform using an oscilloscope ^9^. However, factors such as cable length and probes must be considered during the measurement process, which can easily introduce significant phase errors. Moreover, the sampling rate of the oscilloscope limits the oscillation frequency of the device under test. For high-frequency quadrature oscillator chips, additional mixers are often employed to down-convert the output frequency to a lower frequency before measuring phase accuracy with an oscilloscope ^S10^. Nonetheless, due to large frequency fluctuations, this method suffers from low measurement resolution.

**2) Indirect measurement using image rejection ratio**

This method involves integrating a single-sideband (SSB) mixer ^S11^ or a reflective-type modulator ^S12^ within a multi-phase oscillator, and extracting phase error by measuring the image rejection ratio. However, this approach requires precise symmetric layout and interconnections to minimize phase uncertainty.

**3) Direct measurement using a VNA**

The receiver mode of a Vector Network Analyzer (VNA) can directly measure the phase difference between two ports ^17, 47, 48^. The test can eliminate the effect of cables, probes, and some other factors on the phase through TRL calibration, power calibration, and other error correction techniques, with a phase deviation of 0.1° or less.

**References:**

S1 Shen, G., Zhang, C., Feng, W. & Che, W. Millimeter-wave low-loss on-chip metamaterial for 5G communication based on non-periodic composite right-/left-handed transmission line. *Applied Physics Letters* **122**, 211704 (2023).

S2 Zhou, J., Rao, Y., Yang, D., Qian, H. J. & Luo, X. Compact wideband BPF with wide stopband using substrate integrated defected ground structure. *IEEE Microw. Wireless Compon. Lett.* **31**, 353–356 (2021).

S3 He, J., Wei, Y., Gong, Y., Wang, W. & Park, G.-S. Investigation on a W band ridge-loaded folded waveguide TWT. *IEEE Trans. Plasma Sci.* **39**, 1660–1664 (2011).

S4 Lu, Z. *et al.* High power folded waveguide traveling wave tube based on variable-width technology. *Physics of Plasmas* **26**, 053106 (2019).

S5 Jing-Yu, G. *et al.* 340 GHz folded diamond shaped waveguide slow-wave structure.

S6 Atasoyu, M., Metin, B., Kuntman, H. & Cicekoglu, O. Simple realization of a third order butterworth filter with MOS-only technique. *AEU - International Journal of Electronics and Communications* **81**, 205–208 (2017).

S7 O’Mahony, F., Yue, C. P., Horowitz, M. A. & Wong, S. S. A 10-GHz global clock distribution using coupled standing-wave oscillators. *IEEE J. Solid-State Circuits* **38**, 1813–1820 (2003).

S8 Mirzaei, A. ., Heidari, M. E., Bagheri, R. ., Chehrazi, S. . & Abidi, A. A. The quadrature LC oscillator: A complete portrait based on injection locking. *IEEE J. Solid-State Circuits* **42**, 1916–1932 (2007).

S10 Tillman, F., Troedsson, N. & Sjland, H. A 1.2 volt 1.8GHz CMOS quadrature front-end. in *2004 Symposium on VLSI Circuits. Digest of Technical Papers (IEEE Cat. No.04CH37525)* 362–365 (Widerkehr and Associates, Honolulu, HI, USA, 2004). doi:10.1109/VLSIC.2004.1346616.

S11 Guermandi, D., Tortori, P., Franchi, E. & Gnudi, A. A 0.83-2.5-GHz continuously tunable quadrature VCO. *IEEE J. Solid-State Circuits* **40**, 2620–2627 (2005).

S12 Chang, H. *et al.* A 45-GHz quadrature voltage controlled oscillator with a reflection-type IQ modulator in 0.13-μm CMOS technology. in *2006 IEEE MTT-S International Microwave Symposium Digest* 739–742 (IEEE, San Francisco, CA, 2006). doi:10.1109/MWSYM.2006.249758.
